# Targeted Epigenetic Interventions in Cancer with an Emphasis on Pediatric Malignancies

**DOI:** 10.3390/biom13010061

**Published:** 2022-12-28

**Authors:** Zsuzsanna Gaál

**Affiliations:** Department of Pediatric Hematology-Oncology, Institute of Pediatrics, University of Debrecen, 4032 Debrecen, Hungary; zsuzsanna.gaal.46@gmail.com

**Keywords:** pediatric cancer, epigenetics, hypomethylating agents, histone modifiers, nucleosome remodeling, clinical translation

## Abstract

Over the past two decades, novel hallmarks of cancer have been described, including the altered epigenetic landscape of malignant diseases. In addition to the methylation and hyd-roxymethylation of DNA, numerous novel forms of histone modifications and nucleosome remodeling have been discovered, giving rise to a wide variety of targeted therapeutic interventions. DNA hypomethylating drugs, histone deacetylase inhibitors and agents targeting histone methylation machinery are of distinguished clinical significance. The major focus of this review is placed on targeted epigenetic interventions in the most common pediatric malignancies, including acute leukemias, brain and kidney tumors, neuroblastoma and soft tissue sarcomas. Upcoming novel challenges include specificity and potential undesirable side effects. Different epigenetic patterns of pediatric and adult cancers should be noted. Biological significance of epigenetic alterations highly depends on the tissue microenvironment and widespread interactions. An individualized treatment approach requires detailed genetic, epigenetic and metabolomic evaluation of cancer. Advances in molecular technologies and clinical translation may contribute to the development of novel pediatric anticancer treatment strategies, aiming for improved survival and better patient quality of life.

## 1. Introduction

**Figure 1:**
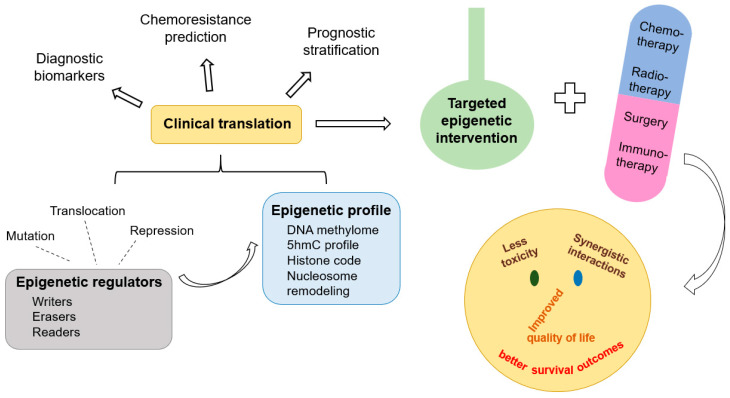
Epigenetic agents in the landscape of personalized anticancer treatment.

The five year overall survival (OS) of children diagnosed with cancer increased from 58% to 84.7% between the mid-1970s and mid-2010s, respectively [1,2]. The cancer mortality rate among pediatric and adolescent patients has declined by more than 50% over the last 50 years [2]. However, chemoresistance and toxicity are still responsible for many deaths. Epigenetic alterations are heritable, and reversible changes in gene expression pattern [3] offer promising opportunities for clinical translation (Figure 1).

Mutations and translocations of genes encoding epigenetic enzymes and histone proteins are frequently identified in cancer, such as TET2 and DNMT3A mutations, translocations of MLL and NUP98 genes in acute myeloid leukemia (AML), mutations of ARID1A and SMARCA4 genes in gliomas, SETD2 mutations in clear cell renal cell carcinoma (ccRCC), and oncohistone mutations in pediatric brain and bone tumors [4,5,6,7,8,9,10,11,12,13,14,15]. Malignant diseases also feature alterations in their epigenetic profiles and altered expression of epigenetic modifiers [16,17,18,19], which can be applied as biomarkers in differential diagnosis, chemoresistance prediction and risk stratification [20] (Table 1). 

According to DNA-methylation profiles, T-cell lymphoblastic lymphoma and pilocytic spinal cord astrocytoma can be distinguished from T-ALL and diffuse leptomeningeal glioneuronal tumors, respectively [21,22]. The methylation level of TFAP2A in circulating tumor DNA is a diagnostic biomarker for retinoblastoma [23]. Differentially methylated positions are promising markers for platinum chemotherapy resistance in various cancers [24]. The expression level of SIRT2 has a positive relationship with cytosine arabinoside and daunorubicin resistance in AML cells [25]. The activation of the EZH2-Stat3 signaling axis is implicated in the development of temozolomide resistance in glioblastoma [26]. The loss of TET2 and reduced levels of genomic 5-hydroxymethylcytosine (5hmC) are associated with poor survival in AML [27]. NUP98/NSD1 fusion is strongly associated with adverse prognosis in pediatric AML [28]. In juvenile myelomonocytic leukemia (JMML), a high level of DNA methylation indicates high relapse incidence and inferior OS, while a low methylation level is associated with a favorable outcome [29]. EZH2 mutation and SETD2 deficiency correlates with poor survival in myelodysplastic syndrome (MDS) [30,31]. A low level of SIRT6 predicts poor relapse-free survival (RFS) in Hodgkin lymphoma [32]. A high level of methylated O^6^-methylguanine-DNA methyltransferase promoter methylation is associated with a favorable outcome of medulloblastoma [33], loss of 5hmC correlates with adverse prognosis in WHO grade II diffuse astrocytomas [34], while EZH2 expression has been described as an independent marker of poor prognosis in pediatric ependymoma [35]. 5hmC profiles correlate with metastatic burden in neuroblastoma [36]. Higher levels of DOT1L expression are associated with poor OS and RFS in ccRCC [37]. The DNA methylation profile has been described as an independent prognostic biomarker for pediatric adrenocortical tumors [38].

In this review, possibilities of targeted epigenetic interventions are discussed in the most common pediatric malignancies, including acute leukemias, lymphomas, neuroblastoma, gliomas, soft tissue sarcomas and kidney tumors (Table 2). The vast majority of cited publications and clinical trials summarize experiences with pediatric and adolescent patients. However, since clinical experience can be first obtained in adult cancer patients (following successful in vitro and in vivo experiments), some data originating from cell lines, animal models and adult patients are also included in order to highlight the wide variety of novel opportunities. Major groups of epidrugs are discussed based on the epigenetic modifications that they target. 

## 2. DNA Methylation

Methylation of the fifth carbon of cytosines is catalyzed by DNA methyltransferase (DNMT) enzymes, resulting in the formation of 5-methylcytosine (5mC) and transcriptional repression [39]. DNMT1 and DNMT3 are canonical DNMTs that catalyze maintenance and de novo DNA methylation, respectively [40]. Azacitidine and 5-aza-2′-deoxycytidine (decitabine) are cytosine analogs, commonly referred to as hypomethylating agents (HMA) that inhibit DNMT enzymes [41].

### 2.1. HMA and Hematological Malignancies

Azacitidine and decitabine have been approved for therapy of MDS and AML since 2004 and 2006, respectively [42]. According to single-center pediatric experience with venetoclax+azacitidine treatment, morphologic response and MRD negativity have been achieved in 2/2 high-grade MDS and 4/6 AML patients, respectively [43]. Compared to AML-type chemotherapy, a much better survival rate was registered with a decitabine-combined minimally myelosuppressive regimen bridged with allo-HSCT in children with advanced MDS [44]. Successful administration of HMAs have been reported in the case of three adolescent AML patients with monosomy 5/del(5q), who received decitabine treatment during both remission induction and conditioning, and were free of disease at 3.6, 3.2, and 3.0 years after HSCT, respectively [45]. According to a phase 1 clinical trial (NCT01861002) published in 2018, azacitidine can be used safely in sequence with intensive chemotherapy in relapsed/refractory pediatric AML and offers encouraging clinical activity [46]. An infant with JMML, harboring somatic KRAS mutation and monosomy 7, was reported in 2019 who achieved sustained remission following azacitidine monotherapy [47]. An adolescent patient with chronic myelomonocytic leukemia (CMML) received venetoclax/decitabine treatment as a bridge to HSCT [48]. Decitabine has shown antineoplastic activity in ALK+ anaplastic large cell lymphoma (ALCL) cell lines [49]. Moreover, according to in vitro and in vivo results, low-dose crizotinib with decitabine treatment completely suppressed the emergence of resistant cells in ALK+ ALCL [50].

### 2.2. HMA and Solid Tumors 

According to recently published data, DNMT3A inhibitor SGI-1027 repressed the development of glioblastoma by indirect inhibition of the TGF-β signaling pathway [51]. In relapsed or progressive rhabdoid tumors, radiological signs of antitumor activity have been registered following decitabine-augmented chemotherapy [52]. Decitabine was found to potentiate the cytotoxic effects of cisplatin in neuroblastoma cells by the induction of RIG-I-related innate immune response [53]. HMA treatment with decitabine increased the susceptibility of rhabdomyosarcoma, Ewing sarcoma and osteosarcoma cell lines to cytotoxic T-lymphocyte mediated lysis [54]. Furthermore, treatment of 143B osteosarcoma cells with decitabine resulted in the inhibition of osteosarcoma growth and metastasis by enhanced expression of ERα [55]. In synovial sarcoma cell lines, decitabine is also suggested to have good therapeutic potential [56]. The Ras association domain-containing protein 1 isoform A (RASSF1A) promoter hypermethylation might be involved in the development and aggressiveness of some pediatric renal tumors and correlated with a poor prognosis. Hypermethylation in the RASSF1A promoter region in rhabdoid tumor of the kidney and ccRCC is associated with aggressive disease and poor prognosis, which could successfully be reversed by the administration of decitabine treatment [57].

## 3. Targeting DNA-Hydroxymethylation

TET proteins are methylcytosine dioxygenase enzymes that catalyze the oxidation of 5mC to 5-hydroxymethylcytosine (5hmC) [58]. TET2 is a master epigenetic regulator of hematopoiesis whose function is inhibited by the recurrent mutations of isocitrate dehydrogenase (IDH) enzymes, resulting in the formation of the oncometabolite 2-hydroxyglutarate [59]. Therefore, enhancing TET2 enzymatic activity or restoring *TET2* transcription may be clinically beneficial in hematological malignancies [5]. On the other hand, the TET1/2 inhibitor (Bobcat339) has been shown to reduce T-ALL burden by targeting the dependence of T-lymphoblasts on the tricarboxylic acid cycle for their growth and survival [60,61]. In glioma cell lines, overexpression of TET3 partially restored the genome-wide 5hmC patterns of control brain samples [62]. Pharmacological inhibition of TET1 reduced cell viability in a mouse model of medulloblastoma [63]. In osteosarcoma cells, enhanced expression of TET1 was associated with an increase in apoptosis rate [58], while the inhibition of TET2-dependent induction of IL-6 is a potential therapeutic approach through antagonizing metastasis formation [64].

## 4. Histone Code

Nucleosomes are composed of an octamer of four core histones (H3, H4, H2A and H2B), wrapped with 147 base pairs of DNA [65]. N-terminal histone tails are enriched with a variety of posttranslational modifications (PTMs) [66]. Histone modifications may result in both activation and repression of transcription, controlled by an array of histone modifiers. The balance between activating and repressing histone marks is commonly disrupted in malignant diseases that can be therapeutically targeted.

### 4.1. Histone Acetylation

Histone acetyltransferase (HAT) enzymes acetylate ε-amino groups of histone lysine residues by three major HAT families, namely p300/CREB-binding protein (p300/CBP), MYST (Moz, Ybf2, Sas2 and Tip60) and GNAT (GCN5-related N-acetyltransferase) [67], described as critical regulators of cell development and carcinogenesis [68]. Histone lysine acetylation results in the activation of transcription.

GCN5 inhibitor α-methylene-γ-butyrolactone 3 decreased acetylation and protein level of the chimeric transcription factor E2A-PBX1 in pediatric pre-B-cell ALL with t(1;19) translocation [69]. In non-APL AML, GCN5 was described as a potential therapeutic target through its contribution to ATRA resistance via aberrant acetylation of histone 3 lysine 9 (H3K9ac) residues [70]. Inactivation of MOF enzyme (MYST1) suppressed leukemia development in a NUP98-HOXA9-driven AML model [71]. Activators of p300 can be applied in the treatment of MDS with TET2 inactivating mutations in order to suppress its transition to AML [72]. In Burkitt lymphoma, inhibition of GCN5 attenuated BCR signaling and reduced the tumorigenic properties of cells [73]. Epigallocatechin-3-gallate blocks p300-mediated acetylation of p65 protein, thereby impairing the transformation of B-cells by EBV [68]. 

Caspase-independent cell death was triggered in neuroblastoma cell culture by PU139 (pan-inhibitor of HAT enzymes) [74]. Silencing or pharmacological inhibition of PCAF in alveolar rhabdomyosarcoma resulted in the down-regulation of PAX3-FOXO1 reduced proliferation and tumor burden in xenograft mice models [75]. Inhibition of the HBO enzyme (MYST2) via intraperitoneal injection of a single dose of WM-3835 potently suppressed the growth of an osteosarcoma xenograft in SCID mice [76]. Tip60 is a novel therapeutic target in osteosarcoma by promoting the effects of KDM2 acetylation on proliferation and metastasis formation of tumor cells [77].

### 4.2. Histone Deacetylation

According to their sequence similarities with yeast enzymes, 18 human zinc-dependent histone deacetylases (HDACs) have been identified [78,79]. The first clinically successful HDAC inhibitor, suberoylanilide hydroxamic acid (SAHA/vorinostat), was approved by the FDA in 2006 as a treatment for refractory or relapsed cutaneous T-cell lymphoma [80]. 

#### 4.2.1. HDACi and Hematological Malignancies

According to a phase 1 clinical trial that was performed with panobinostat in 2020 (NCT02676323), 8/17 pediatric patients with relapsed or refractory (R/R) AML achieved complete remission (CR), and no dose-limiting toxicities were observed [81]. In a phase I trial (NCT02412475) published in 2022, decitabine and vorinostat was well-tolerated and effective in R/R pediatric AML patients in combination with fludarabine, cytarabine and G-CSF (FLAG) treatment [82]. In the MLL-AF9 driven MOLM-13 cell line, mocetinostat reduced cell viability and induced apoptosis [83], while HDAC inhibitor I13 could be a potent and selective agent in AML patients with t(8;21) translocation or MLL-rearrangement to surmount differentiation block [84]. Belinostat induced granulocytic differentiation of acute promyelocytic leukemia HL-60 cells more effectively compared with retinoic acid treatment alone, that was associated with histone H4 hyperacetylation of the C/EBPα promoter region [85]. In t(4;11)-positive primary infant ALL cells, HDAC inhibitors including trichostatin A, vorinostat, panobinostat, valproic acid and romidepsin effectively induced leukemic cell death accompanied by the downregulation of MYC proto-oncogene as well as the MLL-AF4 fusion product [86]. In a large phase II clinical study (NCT00742027) in patients with relapsed classical Hodgkin’s lymphoma, panobinostat reduced tumor measurements in 74% of patients, including 23% partial remissions and 4% CR [87]. Romidepsin has been shown to potentiate the anti-tumor effect of anti-CD20 chimeric antigen receptor (CAR) modified expanded peripheral blood natural killer (NK) cells against rituximab-sensitive and -resistant Burkitt lymphoma in immunodeficient mice [88]. Cay10603, a potent HDAC6 inhibitor inhibited cell cycle progression in Burkitt lymphoma cell lines [89].

#### 4.2.2. HDACi and Solid Tumors

HDACi SAHA, sodium butyrate and trichostatin A induced apoptosis related to dissipation of mitochondrial membrane potential and activation of caspase enzymes in Daoy and UW228-2 medulloblastoma cells [90]. HDAC6-selective inhibitors demonstrated in vitro therapeutic potential against group 3 medulloblastoma [91]. Antiproliferative effects of vorinostat and mocetinostat were described in different human glioma cells [92]. Unfortunately, vorinostat failed to improve outcome in childhood diffuse intrinsic pontine glioma (DIPG) [93]. On the other hand, treatment of group C ependymoma DKFZ-EP1NS cells with vorinostat induced neuronal differentiation and loss of stem cell-specific properties [94]. OBP-801 (spiruchostatin A) induced G2/M phase arrest and suppressed tumor growth in human neuroblastoma cells and in a mouse xenograft model, respectively [95]. Selective dose-dependent cytotoxicity of romidepsin (depsipeptide) was described in both single copy and N-myc amplified neuroblastoma cell lines [96]. Panobinostat enhanced NK cell cytotoxicity in soft tissue sarcoma cell lines [97], while targeting HDAC6 is a promising therapeutic option in rhabdomyosarcoma [98]. Selective inhibition of HDAC6 induced the downregulation of EWSR1-FLI1 and significantly reduced its oncogenic functions in Ewing sarcoma cell lines [99]. Pan-HDAC inhibitor LBH589 (panobinostat) treatment resulted in apoptosis induction and inhibition of cell proliferation of SK-NEP-1 and G401 Wilms’ tumor cells. LBH589 also had a significant effect in SK-NEP-1 xenograft tumors [100]. Targeting HDAC1 is a promising therapeutic approach in aggressive hepatoblastoma due to its involvement in the repression of p21 [101]. In retinoblastoma, intravitreal belinostat was equally effective as standard-of-care melphalan but without retinal toxicity [102]. CUDC907, a dual phosphoinositide-3 kinase/HDAC inhibitor, promoted apoptosis of NF2 schwannoma cells [103].

### 4.3. Sirtuin Enzymes

Sirtuin (SIRT) enzymes compose an NAD^+^-dependent class III subfamily of HDAC enzymes with characteristic intracellular localizations and variable enzyme activities that are also key players in cancer cell metabolism [104]. Seven members of the family have been identified (SIRT1-7), among which SIRT1 was the first sirtuin to be shown to be involved in cancer [105].

Although tenovin-6-mediated SIRT1/2 inhibition hampered the growth of primary cells from children with ALL [106], and silencing of SIRT1 prolonged the lifespan in a mouse model of T-ALL [107], the growth of leukemic cells was promoted by SIRT1 inhibition and reduced by SIRT1 activation in two T-ALL cell lines carrying Notch mutations [108]. Inhibition of SIRT1 reduced proliferation of AML cell lines with t(8;21) translocation [109]. NRD167, an inhibitor of SIRT5 enzyme, reduced glutamine utilization and induced apoptosis in primary AML samples and cell lines [110]. Downregulation of the SIRT1 protein has been described in MDS stem and progenitor cells, whereas activation of SIRT1 represents a promising means to target MDS [111]. Inhibition of SIRT1 deprived Burkitt lymphoma cells of their most important survival signal by reducing MYC protein levels [112].

In patient-derived IDH mutant glioma lines, overexpression of SIRT1 led to inhibition of cell growth [113]. On the other hand, specific inhibition of SIRT1 by EX527 induced cell apoptosis in two glioma cell lines by activating p53 [114]. Based on its interaction with the ERK/STAT3 signaling pathway, SIRT7 may also function as a valuable target for the treatment of human glioma [115]. Downregulation of SIRT3 was associated with suppression of growth and migration of glioblastoma cells [116]. Overexpression of SIRT4 significantly reduced proliferation [117], while inhibition of SIRT6 induced differentiation of neuroblastoma cell lines [118]. Pharmacological inhibition of SIRT1 and SIRT2 impaired autophagy process and induced cell death in pediatric soft tissue sarcoma cell lines [119]. SIRT1i molecules are promising therapeutic targets to treat metastatic disease in Ewing sarcoma by mediating tumor suppressive Notch response [120]. In ccRCC cells, overexpression of SIRT4 reduced proliferation and migration [121], while inhibition of SIRT6 may counteract the Bcl-2-dependent pro-survival pathway [122]. Overexpression of SIRT6 induced apoptosis of nasopharyngeal carcinoma cells by inhibiting NFκB signaling [123].

### 4.4. Histone Methylation

Histone methylation occurs on arginine and lysine residues, resulting in different outcomes for transcriptional regulation [66]. Lysines can be mono-, di- or trimethylated on their ε amine group, arginines can be monomethylated (MMA), symmetrically dimethylated (SDMA) or asymmetrically dimethylated (ADMA) on their guanidinyl group, the processes of which are believed to turnover more slowly than many other PTMs [124]. Three families of “writer” histone methyltransferases are distinguished based on their domain structure and the methylated residue [125]. Vast majority of enzymes that catalyze histone lysine methylation (KMT) contain the so-called SET domain, except for the DOT1L enzyme. The third group is composed of histone arginine methyltransferase PRMT enzymes. Based on the amount of recently published results, EZH2 is discussed separately from other SET-domain containing KMT enzymes.

#### 4.4.1. EZH2i

EZH2i 3-deazaneplanocin A induced growth inhibition and increased apoptotic rate in T-ALL Jurkat cells [126]. EZH1/2 dual inhibitor, DS-3201 inhibited the growth of B-ALL, harboring MLL-AF4 significantly in a patient-derived xenograft mouse model [127]. Proliferation of pediatric acute monocytic leukemia cells was inhibited by targeting EZH2-mediated methylation of histone H3 [128]. Targeting EZH2 in myelodysplasia is a promising treatment strategy, since it promoted the transformation from MDS to AML [129]. Tazemetostat (EPZ-6438) lead to potent antitumor activity in preclinical models of EZH2-mutant non-Hodgkin lymphoma (NHL) [130]. In a first-in-human, open-label, phase 1 study (NCT01897571), published in 2018, tazemetostat showed a favorable antitumor activity in refractory B-cell NHL and advanced solid tumors [131]. Valemetostat is the first dual EZH1/2 inhibitor, approved for the treatment of adult T-cell lymphoma in September 2022 [117].

Pharmacological inhibition of EZH2 impaired proliferation and induced apoptosis of SHH medulloblastoma cells in vitro [132]. Based on its interaction with the tumor suppressor protein p16, EZH2 is a potential therapeutic target for H3K27M-mutant pediatric gliomas [133]. EZH2i GSK343 led to significantly decreased viability, migration and invasion in neuroblastoma cell lines [134], while EPZ005687 reduced cell viability and colony formation in rhabdomyosarcoma cell lines [135]. Based on the positive results of a single-arm phase II basket study (NCT02601950), the FDA granted accelerated approval of tazemetostat in 2020 for patients aged 16 years and older with metastatic or advanced epithelial sarcoma not eligible for complete resection [136]. EZH1/2 dual inhibitors are promising therapeutic strategies for pediatric malignant rhabdoid tumors [137].

#### 4.4.2. Other SET-Domain-Containing KMT Enzymes

Covalent inhibition of the NSD1 enzyme impaired colony formation of primary AML cells harboring t(5;11) translocation and NUP98-NSD1 fusion that is predominantly observed in pediatric AML patients [28,138]. In childhood ALL cells, inhibition of G9a (EHMT2) with BIX01294 abrogated transendothelial migration [139]. In cultured cells of AML and ALL, inhibition of G9a reduced cell proliferation and promoted apoptosis [140]. Inhibition of SUV39H1 reduced proliferation and cell migration in pediatric astrocytoma cell lines [141]. In pediatric-type high-grade glioma cells, inhibition of SUV39H1 and SUV39H2 methyltransferases was confirmed to be lethal [142]. Treatment of neuroblastoma cells with BIX01294 resulted in the inhibition of cell growth and proliferation [143]. G9a was also described as a potential therapeutic target in embryonal rhabdomyosarcoma due to its interaction with Wnt signaling [144]. Inhibition of G9a reduced metastatic development in mice models of Ewing sarcoma [145]. Knockdown or inhibition of SUV39H1 suppressed the growth of ccRCC cells by inducing ferroptosis [146].

#### 4.4.3. DOT1L

DOT1L is the only KMT enzyme that does not contain a SET domain and specifically targets histone H3 lysine 79 (H3K79) residues for mono-, di- or trimethylation [147]. MLL-rearranged acute leukemias are confirmed to be dependent on aberrant H3K79 methylation by the DOT1L enzyme [148]. Inhibition of DOT1L with pinometostat (EPZ5676) resulted in significant differentiation effects in MLL-fused leukemia cell lines [149]. Based on the results of a phase 1 clinical trial in pediatric R/R leukemia patients with MLL-rearrangement, performed between 2014 and 2016 (NCT02141828), administration of EPZ5676 in combination with other antileukemia agents is warranted [150]. In vivo efficacy of DOT1L inhibition has also been observed in a nude rat xenograft model of *DNMT3A*-mutant AML [151]. The inhibition of DOT1L with SGC0946 reduced H3K79 methylation and proliferation of N-Myc amplified neuroblastoma cells [152]. In murine orthotopic xenografts of retinoblastoma, EPZ5676 significantly improved treatment efficacy [153].

#### 4.4.4. PRMT Enzymes

Among the nine PRMTs described, type I, II and III enzymes are able to generate ADMA, SDMA or MMA, respectively [154]. Maintenance of FLT3-ITD AML cells was markedly blocked by genetic or pharmacological inhibition of PRMT1 enzyme [155]. Moreover, inhibition of PRMT1 with MS023 abolished arginine methylation of FLT3 and disrupted the maintenance of MLL-rearranged ALL cells [156]. Spliceosomal mutant leukemias were found to be preferentially sensitive to PRMT inhibition [157]. 28d, a potent inhibitor of type I PRMTs, effectively inhibited cell proliferation in several types of leukemia cell lines [158]. Inhibition of PRMT5 induced cell death in different types of NHL cell lines through the abrogation of proliferation-related signaling pathways [159]. The depletion of PRMT1 induced apoptosis in medulloblastoma cells [160], while treatment with PRMT5 inhibitor decreased tumor growth and increased survival in a SHH medulloblastoma mouse model [161]. AMI-1 and SAH, pan-inhibitors of PRMT enzymes, decreased cell viability and reduced the invasive phenotype of rhabdomyosarcoma cells [162].

### 4.5. Histone Demethylation

Histone lysine demethylases are categorized into two subgroups, KDM1-family (KDM1A = LSD1 and KDM1B) and Jumonji C (JmjC) domain-containing histone demethylase enzymes (JHDMs) [163]. Arginine demethylation occurs via peptidyl arginine deiminase 4 by converting arginine to citrulline [164].

#### 4.5.1. KDM1-Family 

The knockdown or chemical inhibition of LSD1 dominated C/EBPα instead of the GATA1 transcription factor, resulting in metabolic shifts and growth arrest in erythroleukemia cells [165]. Inhibition of LSD1 by the highly potent FY56 compound induced differentiation in MOLM-13 and MV4-11 AML cell lines [166]. LSD1 inhibitor S2157 has been confirmed to efficiently pass through the blood–brain barrier and eradicate CNS leukemia in T-ALL mice models [167]. According to xenograft studies with patient-derived Ewing sarcoma cell lines, LSD1 inhibitor HCI2509 disrupted the oncogenic transcriptional activity of EWS/ETS fusion proteins [168]. In retinoblastoma cells, inhibition of LSD1 by SP2509 resulted in growth inhibition via the suppression of β-catenin pathway [169].

#### 4.5.2. JHDM Enzymes

According to in vitro and in vivo biological function experiments, KDM4A (JMJD2A) inhibitor SD49-7 suppressed the progression of leukemia stem cells through the activation of the apoptosis signaling pathway [170]. GSKJ4, an inhibitor of KDM6A (UTX) and KDM6B (JMJD3) enzymes, induced apoptosis and cell-cycle arrest in Kasumi-1 cells, decreased proliferation of U-937 and K-562 cells, and attenuated disease progression in a human AML xenograft mouse model [171,172]. Inhibition of KDM3C (JMJD1C) by JDI-10 decreased lipid synthesis-associated genes and induced apoptosis in MLL-rearranged AML cells [173]. Inhibition of KDM5A (JARID1A) greatly potentiated the differentiation of APL cell line NB4 [174]. Genetic and pharmacologic inhibition of KDM4B (JMJD2B) substantially delayed tumor growth in preclinical subcutaneous xenograft models of PAX3-FOXO1-driven alveolar rhabdomyosarcoma [175]. Depletion of KDM3A (JHDM2A) also inhibited growth and metastasis formation of the oncofusion-positive rhabdomyosarcoma cells in vivo [176]. KDM5B (JARID1B) inhibitor AS-8351 suppressed proliferation and induced cell cycle arrest in Ewing sarcoma cell lines [177]. Knockdown of KDM3A suppressed aerobic glycolysis and weakened the growth of osteosarcoma cells in vitro and in a nude mouse model [178]. Downregulation of JMJD6 enzyme resulted in impaired colony formation of ccRCC cells [179]. SMARCA4-deficient tumors are confirmed to be strongly dependent on KDM6A and KDM6B histone demethylases, which are also novel promising therapeutic targets [180].

## 5. Reader Molecules

**Figure 2:**
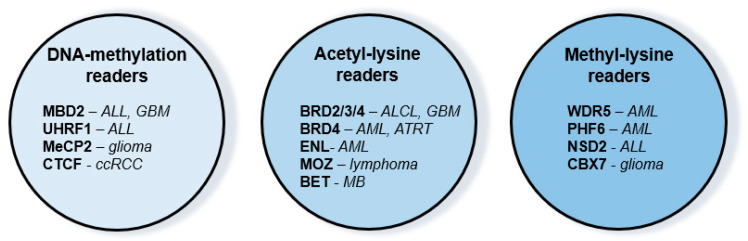
Reader molecules as novel therapeutic targets in pediatric malignancies (see references in the text). Abbreviations of diseases: ALCL: anaplastic large cell lymphoma, ALL: acute lymphoblastic leukemia, AML: acute myeloid leukemia, ATRT: atypical teratoid rhabdoid tumor, ccRCC: clear cell renal cell carcinoma, GBM: glioblastoma multiforme, MB: medulloblastoma.

Numerous chromatin-associated factors can specifically interact with methylated CpG dinucleotides and modified histones via distinct domains, which are essential for the assembly of multiprotein epigenetic regulator complexes. Over the previous decade, growing numbers of reports were published about successful inhibition of so-called reader proteins in cancer (Figure 2).

### 5.1. DNA Methylation Readers

Methyl-CpG binding zinc finger proteins, MBD-containing proteins and SRA domain-containing proteins are the three subgroups of methyl-binding proteins (MBPs), among which methyl-CpG-binding protein 2 (MeCP2) was the first MBD-containing protein discovered in 1992 [181,182]. Deletion of MBD2 effectively switched off the abnormal activation of Wnt signaling in T-ALL cell lines and mice models [183]. Knockdown of UHRF1 protein reduced c-Myc protein expression and cell viability in both B-ALL and T-ALL in vitro [184]. Overexpression of MeCP2 in C6 glioma cells resulted in decreased proliferation, migration and invasion [185]. In primary glioblastoma tumor samples, knockdown of MBD2 restored expression of the tumor suppressor BAI1 protein [186]. According to functional experiments, overexpression of CTCF could inhibit migration and invasion of ccRCC cells [187].

### 5.2. Acetyl-Lysine Readers

Reader proteins of lysine acetylation contain bromodomain (BRD), double plant homeodomain (PHD) fingers or a YEATS (*Yaf9*, ENL, AF9, Taf14, Sas5) domain, among which bromodomain and extra terminal (BET) proteins compose a distinct subfamily of BRD group [188,189,190]. Inhibition of BRD4 by I-BET151 efficiently blocked proliferation of AML cells in primary murine hematopoietic stem and progenitor cells harboring t(10;17)(p15;q21) translocation [191]. TDI-11055, an orally bioavailable small-molecule inhibitor of ENL, blocked disease progression in patient-derived xenograft models of NPM1-mutated and MLL-rearranged AML [192]. Inhibition of MOZ (MYST3) arrested tumor growth and induced senescence in mice models of lymphoma [193]. Bromodomain inhibitor OTX015 led to downregulation of MYC and cell cycle arrest in ALK+ ALCL cells [194]. BET inhibitor JQ1 was found to potently decrease viability of MYC-amplified medulloblastoma cells [195]. In mouse glioblastoma cells, OTX015 showed much higher antiproliferative effect compared to that of JQ1 [196]. Targeted inhibition of BRD4 reduced cell proliferation and invasiveness of ATRT cell lines [197]. OTX015 treatment resulted in reduced proliferation and upregulation of apoptosis-related proteins in pediatric patient-derived ependymoma stem cell models [198].

### 5.3. Methyl-Lysine Reader Domains

Major families of methyl-lysine reader domains include chromodomains (CD), PHD, WD40 repeat (WDR) and PWWP (proline–tryptophan–tryptophan–proline) domains [199]. CD inhibitor SW2_110A inhibited proliferation of THP1 leukemia cells [200]. Deletion of WDR5 impaired colony forming ability of MLL-AF9 positive cells in a murine leukemia model [201]. PHF6 and NSD2 have been described as promising therapeutic targets in PHF6-mutant AML and ALL, respectively [202,203]. Overexpression of CBX7 inhibited cell proliferation, migration and colony formation of glioma cell lines [204].

## 6. Targeting Nucleosome-Remodeling Machinery

**Figure 3:**
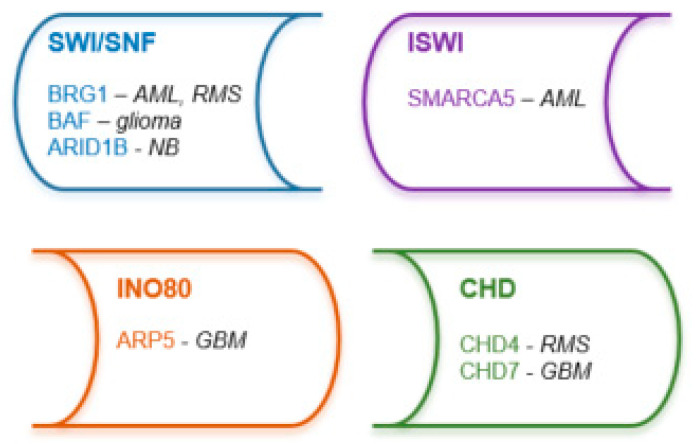
Targetable components of nucleosome remodeling complexes in pediatric malignancies (see references in the text). Abbreviations of diseases: AML: acute myeloid leukemia, GBM: glioblastoma multiforme, NB: neuroblastoma, RMS: rhabdomyosarcoma.

Four major subfamilies of chromatin remodeling complexes have been identified, switch/sucrose non-fermentable (SWI/SNF), imitation SWI (ISWI), chromodomain-helicase DNA-binding protein (CHD) and inositol-requiring mutant 80 (INO80), which are responsible for mobilization of nucleosomes at target-promoters and -enhancers to modulate gene expression (Figure 3) [10,205,206,207].

Dual inhibitors of SWI/SNF catalytic subunits, BRM (SMARCA2) and BRG1 (SMARCA4) lead to downregulation of leukemic pathway genes, including MYC in AML cell lines [208]. Deletion of SMARCA5, catalytic subunit of ISWI complex, resulted in karyorrhexis and blocked cell cycle progression in AML cell lines [209]. BAF components of SWI/SNF complex have been confirmed to maintain an oligodendrocyte precursor cell (OPC)-like state in glioma stem cells, thereby providing novel candidates for targeted therapy in H3K27M-mutant gliomas [210]. CHD7 knockout inhibited tumor growth in an orthotopic mouse xenograft model of glioblastoma [211]. ARP5, component of INO80 complex, has also been described as a novel therapeutic target in glioblastoma due to its suggested oncogenic role in the disease [212]. Inhibition of BRG1 resolved differentiation blockade in fusion positive rhabdomyosarcoma cell lines [213], while depletion of CHD4, a coregulator of the oncogenic PAX3-FOXO1 transcription factor, resulted in reduced viability of fusion-positive but not of fusion-negative rhabdomyosarcoma in vitro [214]. ARID1B, a component of the SWI/SNF complex, has been described as a promising novel therapeutic target in ARID1A-mutant neuroblastomas [215].

## 7. Epigenetic Interventions in the Landscape of Anticancer Treatment 

Novel epigenetic drugs have been confirmed to influence the efficacy of other anticancer treatment modalities. Enhanced chemosensitivity, radiosensitivity and improved results of immunotherapy have been described in growing numbers of malignancies co-treated with epigenetic agents.

HMA treatment and depletion of SIRT6 resulted in increased sensitivity of AML cell lines to cytarabine treatment through the restored expression of BIK gene and inhibition of DNA repair process of double-strand breaks, respectively [216,217,218]. Loss of SIRT2 greatly enhanced chemosensitivity of AML cells harboring MLL-ENL fusion protein [219]. Inhibition of the demethylation of KMT enzyme G9a restored sensitivity of treatment-resistant B-ALL to glucocorticoid-induced cell death [220]. HDAC inhibitors may be applied to overcome rituximab resistance in B-cell lymphomas by the upregulation of CD20 expression on lymphoma cells [221]. Inhibition of HAT enzymes P300 and CBP sensitized mantle cell lymphoma to PI3K inhibitor idelalisib treatment in vitro and in vivo [222]. Knockdown of SIRT6 significantly potentiated the efficacy of doxorubicin in osteosarcoma cells [223]. Inhibition of EZH1/2 significantly increased the sensitivity of MYCN-amplified neuroblastoma cells to 5-fluorouracil therapy [224].

Adjuvant administration of decitabine resulted in a radiosynergistic effect in human medulloblastoma cell lines [225], while inhibition of PRMT6 enzyme improved the cytotoxic activity of radiotherapy against glioblastoma stem cells [226]. HMA therapy and entinostat have been described as promising radiosensitizing agents in embryonal rhabdomyosarcoma and PAX3-FOXO1 positive alveolar rhabdomyosarcoma cells, respectively [227,228].

Epidrugs can also prime antitumor immune response, which may give rise to the development of combination strategies with immunotherapy agents [229]. In AML cell lines and primary AML cells, BET inhibition improved antileukemia immunity by regulating PD-1/PD-L1 expression [230]. Decitabine plus anti-PD1 camrelizumab treatment increased the percentage of circulating peripheral central memory T-cells, which correlated with improved clinical response and survival outcome measures in R/R classical Hodgkin lymphoma [231]. Combined HDAC inhibitor and anti-PD-1 antibody treatment significantly promoted tumor regression and improved survival in a murine model of advanced soft tissue sarcoma [232]. Combination of anti-GD2 antibody and vorinostat was found to be highly effective in an aggressive orthotopic neuroblastoma model [233]. According to recently published in vitro results, combination of decitabine with CAR T-cell therapy is an attractive novel therapeutic approach to enhancing the tumor-specific killing of bladder cancer [234].

During the past few years, a growing amount of synergistic interactions of epigenetic agents have been identified. Combining LSD1 and JAK-STAT inhibition exerted synergistic antileukemic effects in Down syndrome-associated myeloid leukemia [235]. Combined treatment of promyelocytic leukemia cell line HL-60 with BRD inhibitor PLX51107 and vorinostat resulted in decreased cell proliferation and dramatically increased apoptotic rate [236]. EZH2 and HDAC inhibitors demonstrated potent synergy in lymphoma cell lines with EZH2 dysregulation [237]. BET-inhibitor JQ1 and CBP-inhibitor ICG-001 treatment synergistically inhibited proliferation and invasion potential in H3K27M-mutated DIPG cell lines [238]. CDK2 inhibitor milcilib synergized with BET inhibitor treatment in group 3 medulloblastoma in vivo and in vivo models [239]. In patient-derived xenograft models of embryonal rhabdomyosarcoma, synergistic growth inhibition was described in case of combinatorial treatment with entinostat and vincristine [240]. According to the results of a systematic review and meta-analysis published in 2018, a combination of HDAC inhibition and HMA therapy does not appear to be more effective and better tolerated than HMA alone in MDS and AML [241].

## 8. Conclusions and Future Perspectives

The major aim of the precision oncology treatment approach in the 21st century is to translate the revolution of molecular and genetic technologies into clinical practice. 

Reversible epigenetic alterations are novel hallmarks of cancer that often develop during early stages of malignant diseases. Due to their widespread interactions and involvement in the regulation of multiple biological processes, disruptions of epigenetic modifiers are considered as central hubs in the pathogenesis of cancer.

Patients should be profiled based on a systematic biological approach when diagnosed with a tumor. In addition to the identification of translocations and molecular genetic alterations, detailed evaluation of the epigenetic profile should be highlighted among future aims that could be completed with metabolomic characterization and the clarification of further, nonmolecular factors such as nutritional status and psychosocial condition.

Although precision medicine has also entered clinics for childhood tumors, major challenges should be noted. Epigenetic profiles of pediatric tumors are markedly different when compared to adult cancers. The biological impact of a certain epigenetic modifier enzyme depends highly on tissue microenvironment. The same chromatin regulator may harbor both tumor suppressant and oncogenic properties depending on the type of tumor. Genetic and epigenetic intratumor heterogeneity represents a remarkable challenge in treatment, contributing to tumor evolution, chemoresistance and relapse [20,237]. Special pharmacokinetic characteristics of different age groups within the pediatric population are to be considered.

Although epigenetic treatments are generally well-tolerated, toxicities and adverse events have also been registered, which are in need of further investigation. Prevention of late toxicities is of distinguished significance in pediatric cancer patients. Ensuring specificity is essential to avoid undesirable side effects, such as the activation of endogenous retroviral elements and long terminal repeats [242]. Azacitidine has been associated with aggravation of disease-associated thrombocytopenia [243], exacerbations of pre-existing crystal-induced arthritis [244] and development of pericardial effusion [245]. Further adverse events during epigenetic treatments have also been described, such as hypertriglyceridemia in a phase 1 study (NCT01321346) with panobinostat in pediatric leukemia and lymphoma patients [246]. Potential development of resistance and impairment of host response against viral replication should be noted [247,248]. 

In summary, targeted epigenetic interventions open new horizons in the treatment of childhood malignancies. Detailed genomic and epigenomic evaluation is required for the administration of patient-tailored combinations of epidrugs and conventional anticancer treatment modalities in the early stage of the disease. However, major challenges have to be resolved, epigenetic agents can contribute to improved survival outcomes, more efficient and less toxic treatment regimens, and improved quality of life for pediatric cancer patients. 

## Figures and Tables

**Table 1:** Examples for epigenetic biomarkers in the differential diagnosis, prognosis and chemoresistance prediction of malignant diseases (see references in the text).

	Diagnosis	Prognosis	Chemoresistance Prediction
**DNA-methylation profile**	T-LBL, pilocytic spinal cord astrocytoma, retinoblastoma	JMML, adrenocortical tumors	Platinum chemotherapy (various cancers)
**TET2**		AML	
**5hmC pattern**	Metastatic neuroblastoma	AML, grade II astrocytoma	
**SIRT2**			Cytosine arabinoside and daunorubicin (AML)
**SIRT6**		Hodgkin lymphoma	
**EZH2**		MDS, ependymoma	Temozolomide (GBM)
**DOT1L**		ccRCC	
**SETD2**		MDS	
**NUP98/NSD1 fusion**		AML	

Abbreviations of diseases: AML: acute myeloid leukemia, ccRCC: clear cell renal cell carcinoma, GBM: glioblastoma multiforme, JMML: juvenile myelomonocytic leukemia, LBL: lymphoblastic lymphoma, MDS: myelodysplastic syndrome.

**Table 2:** Targeted epigenetic interventions in hematological malignancies and solid tumors (see references in the text).

Epigenetic Process	Enzyme Family	Enzyme Subfamily	Enzyme	Agent	Hematological Malignancies	Solid Tumors
**DNA methylation**	**DNMT**		DNMT1,3	Azacitidine, decitabine	MDS, AML, JMML, CMML, ALCL	GBM, neuroblastoma, RMS, synovial src, ccRCC, osteosarcoma, Ewing src, RT
**DNA hydroxymethylation**	**TET**		TET1,2	Bobcat339	T-ALL	Medulloblastoma, osteosarcoma
**Histone acetylation**	**HAT**			PU139 (pan-inhibitor)		Neuroblastoma
		***p300/CBP***	p300, CBP		MDS	
		***MYST***	MOF (MYST1)		NUP98-HOXA9-driven AML	
			HBO (MYST2)	WM-3835		Osteosarcoma
			TIP60			Osteosarcoma
		***GNAT***	GCN5		Pre-B ALL, non-APL AML, Burkitt lymphoma	
			PCAF			Alveolar RMS
**Histone deacetylation**	**HDAC**	**HDAC I, IIa, Iib, IV**	HDAC 1-11			
				Belinostat	APL	Retinoblastoma
				HDAC1i		Hepatoblastoma
				HDAC6i	Burkitt lymphoma	gr3 medulloblastoma, RMS, Ewing src
				I13	t(8;21) AML, MLL-rearranged AML	
				Mocetinostat	MLL-AF9	Glioma
				Panobinostat	R/R AML, relapsed cHL, t(4;11)-positive infant ALL	Soft tissue sarcoma, Wilms’ tumor
				Romidepsin	Burkitt lymphoma, t(4;11)-positive infant ALL	Neuroblastoma
				Spiruchostatin A		Neuroblastoma
				Trichostatin A	t(4;11)-positive infant ALL	Medulloblastoma
				Vorinostat/SAHA	R/R AML, t(4;11)-positive infant ALL	Medulloblastoma, glioma, grC ependymoma
**Histone deacylation**	**SIRT**		SIRT1	Tenovin 6, EX527	ALL, t(8;21) AML, MDS, Burkitt lymphoma	Glioma, soft tissue src, Ewing src
			SIRT2	Tenovin 6	ALL	Soft tissue src
			SIRT3			GBM
			SIRT4			Neuroblastoma, ccRCC
			SIRT5	NRD167	AML	
			SIRT6			Neuroblastoma, ccRCC, nasopharyngeal cc
			SIRT7			Glioma
**Histone Lys methylation**	**KMT**					
		**SET-domain**	EZH2	DZNep, tazemetostat (EPZ-6438), GSK343, EPZ005687	MDS, ALL, monocytic AML, NHL	SHH medulloblastoma, neuroblastoma, H3K27M gliomas, epithelial src, RMS, RT
			G9a (EHMT2)	BIX01294	ALL	Neuroblastoma, embryonal RMS, Ewing src
			NSD1		AML with t(5;11) translocation	
			SUV39H1			Astrocytoma, high-grade glioma, ccRCC
		**w/o SET domain**	DOT1L	Pinometostat (EPZ5676)	MLL-r AML, DNMT3A-mutant AML	Neuroblastoma, retinoblastoma
**Histone Arg methylation**	**PRMT**			AMI-1 and SAH (pan-inhibitors)		RMS
			PRMT1	28d	FLT3-ITD AML, MLL-r ALL	Medulloblastoma
			PRMT5		NHL	SHH medulloblastoma
			PRMT6	EPZ020411		GBM
**Histone demethylation**	**KDM1**		LSD1 (KDM1A)	FY56, S2157, HCI2509, SP2509	AML, T-ALL (CNS)	Ewing src, retinoblastoma
	**JHDM**		KDM3A (JHDM2A)			RMS, osteosarcoma
			KDM3C (JMJD1C)	JDI-10	MLL-r AML	
			KDM4A (JMJD2A)	SD49-7	LSC	
			KDM4B (JMJD2B)			Alveolar RMS
			KDM5A (JARID1A)		APL	
			KDM5B (JARID1B)	AS-8351		Ewing src
			KDM6A (UTX)	GSKJ4	AML	
			KDM6B (JMJD3)	GSKJ4	AML	
			JMJD6			ccRCC

Abbreviations of diseases: ALCL: anaplastic large cell lymphoma, ALL: acute lymphoblastic leukemia, AML: acute myeloid leukemia, APL: acute promyelocytic leukemia, ccRCC: clear cell renal cell carcinoma, CMML: chronic myelomonocytic leukemia, GBM: glioblastoma multiforme, JMML: juvenile myelomonocytic leukemia, LSC: leukemia stem cell, MDS: myelodysplastic syndrome, NHL: non-Hodgkin lymphoma, RMS: rhabdomyosarcoma, RT: rhabdoid tumor, src: sarcoma.

## Data Availability

No new data were created or analyzed in this study. Data sharing is not applicable to this article.

## Abbreviations

5hmC: 5-hydroxymethylcytosine, 5mC: 5-methylcytosine, ADMA: asymmetric dimethylarginine, AML: acute myeloid leukemia, ALCL: anaplastic large cell lymphoma, ALK: anaplastic lymphoma kinase, ALL: acute lymphoblastic leukemia, APL: acute promyelocytic leukemia, ARID1A: AT-rich interactive domain-containing protein 1A, ARP5: actin-related protein 5, ATRA: all-trans retinoic acid, BAF: BRG/BRM-associated factor, Bcl-2: B-cell lymphoma 2, BCR: breakpoint cluster region, BET: bromodomain and extra terminal family, BIK: Bcl-2-interacting killer, BRD: bromodomain, BRG1: Brahma-related gene 1, BRM: Brahma, CAR: chimeric antigen receptor, CBP: CREB binding protein, ccRCC: clear cell renal cell carcinoma, CD: chromodomain, C/EBPα: CCAAT/enhancer-binding protein alpha, CHD: chromodomain-helicase DNA binding protein 5, CNS: central nervous system, CR: complete remission, CTCF: CCCTC-binding factor, DIPG: diffuse intrinsic pontine glioma, DOT1L: disruptor of telomeric silencing 1-like, DNMT: DNA methyltransferase, ERK: extracellular signal-regulated kinase, FDA: Food and Drug Administration, EBV: Epstein-Barr virus, EHMT2: euchromatic histone lysine methyltransferase 2, ERα: estrogen receptor α, EZH2: enhancer of zeste homolog 2, FOXO1: forkhead box protein O1, GCN5: general control non-depressible 5, G-CSF: granulocyte colony-stimulating factor, GD2: disialoganglioside *2,* GNAT: GCN5-related N-acetyltransferase, HAT: histone acetyltransferase, HDAC: histone deacetylase, HMA: hypomethylating agents, HSCT: hematopoietic stem cell transplantation, IDH: isocitrate dehydrogenase, IL-6: interleukin 6, INO80: inositol-requiring mutant 80, ISWI: imitation SWI, JAK: Janus kinase, JARID1A: Jumonji AT-rich interactive domain 1A, JHDM: JmjC domain-containing histone demethylase enzyme, JmjC: Jumonji C domain, JMML: juvenile myelomonocytic leukemia, KDM: histone lysine demethylase, KMT: histone lysine-specific methyltransferase, LSD1: lysine-specific demethylase 1, MBD: methyl-CpG-binding domain, MBP: methyl-binding protein, MDS: myelodysplastic syndrome, MeCP2: methyl CpG binding protein 2, MDM2: mouse double minute 2 homolog, MLL: mixed lineage leukemia, MMA: monomethyl arginine, MOZ: monocytic leukemia zinc finger protein, MRD: minimal residual disease, MYST: Moz, Ybf2, Sas2 and Tip60, NHL: non-Hodgkin lymphoma, NFκB: nuclear factor kappa light chain enhancer of activated B cells, NK: natural killer cell, NPM1: nucleophosmin 1, NSD: nuclear receptor binding SET domain protein, NUP98: nucleoporin 98, OPC: oligodendrocyte precursor cell, OS: overall survival, PAX3: paired box gene 3, PCAF: p300/CBP-associated factor, PD1: programmed cell death 1, PHD: plant homeodomain, PHF6: plant homeodomain finger 6, PRMT: protein arginine methyltransferase, PTM: posttranslational modification, PWWP: proline-tryptophan-tryptophan-proline domain, R/R: relapsed or refractory, RASSF1A: Ras association domain-containing protein 1 isoform A, RFS: relapse-free survival, RIG-I: retinoic acid-inducible gene I, SAHA: suberoylanilide hydroxamic acid, SCID: severe combined immunodeficiency, SDMA: symmetric dimethylarginine, SHH: sonic hedgehog, SIRT: sirtuin, SMARC: SWI/SNF-related matrix associated actin dependent regulator of chromatin, SRA: SET and RING associated domain, STAT: signal transducer and activator of transcription, SUV39H1: suppressor of variegation 3-9 homolog 1, SWI/SNF: switch/sucrose nonfermenting, TFAP2A: transcription factor AP-2 alpha, TET: ten eleven translocation, TGFβ: transforming growth factor-β, UHRF: ubiquitin-like, containing PHD and RING finger domains, UTX: ubiquitously transcribed tetratricopeptide repeat, X chromosome, YEATS: Yaf9 ENL AF9 Taf14 Sas5, WDR: WD40 repeat.

## References

[1] Siegel R.L., Miller K.D., Fuchs H.E., Jemal A. (2021). Cancer Statistics, 2021. CA Cancer J. Clin..

[2] Howlader N., Noone A.M., Krapcho M., Miller D., Brest A., Yu M., Ruhl J., Tatalovich Z., Mariotto A., Lewis D.R. SEER Cancer Statistics Review, 1975–2018.

[3] Kopytko P., Piotrowska K., Janisiak J., Tarnowski M. (2021). Garcinol—A Natural Histone Acetyltransferase Inhibitor and New Anti-Cancer Epigenetic Drug. Int. J. Mol. Sci..

[4] Lio C.-W.J., Yuita H., Rao A. (2019). Dysregulation of the TET family of epigenetic regulators in lymphoid and myeloid malignancies. Blood.

[5] Bensberg M., Rundquist O., Selimović A., Lagerwall C., Benson M., Gustafsson M., Vogt H., Lentini A., Nestor C.E. (2021). TET2 as a tumor suppressor and therapeutic target in T-cell acute lymphoblastic leukemia. Proc. Natl. Acad. Sci. USA.

[6] Liang D.-C., Liu H.-C., Yang C.-P., Jaing T.-H., Hung I.-J., Yeh T.-C., Chen S.-H., Hou J.-Y., Huang Y.-J., Shih Y.-S. (2013). Cooperating gene mutations in childhood acute myeloid leukemia with special reference on mutations of ASXL1, TET2, IDH1, IDH2, and DNMT3A. Blood.

[7] Xu H., Wen Y., Jin R., Chen H. (2022). Epigenetic modifications and targeted therapy in pediatric acute myeloid leukemia. Front. Pediatr..

[8] Ley T.J., Ding L., Walter M.J., McLellan M.D., Lamprecht T., Larson D.E., Kandoth C., Payton J.E., Baty J., Welch J. (2010). *DNMT3A* Mutations in Acute Myeloid Leukemia. N. Engl. J. Med..

[9] Harrison C.J., Hills R.K., Moorman A.V., Grimwade D.J., Hann I., Webb D.K., Wheatley K., de Graaf S.S., Berg E.V.D., Burnett A.K. (2010). Cytogenetics of Childhood Acute Myeloid Leukemia: United Kingdom Medical Research Council Treatment Trials AML 10 and 12. J. Clin. Oncol..

[10] Ciechomska I.A., Jayaprakash C., Maleszewska M., Kaminska B. (2020). Histone Modifying Enzymes and Chromatin Modifiers in Glioma Pathobiology and Therapy Responses. Adv. Exp. Med. Biol..

[11] Qiu L., Hu X., Jing Q., Zeng X., Chan K.M., Han J. (2018). Mechanism of cancer: Oncohistones in action. J. Genet. Genom..

[12] El-Hashash A.H.K. (2021). Histone H3K27M Mutation in Brain Tumors. Adv. Exp. Med. Biol..

[13] Li J., Kluiver J., Osinga J., Westers H., van Werkhoven M.B., Seelen M.A., Sijmons R.H., Berg A.V.D., Kok K. (2016). Functional Studies on Primary Tubular Epithelial Cells Indicate a Tumor Suppressor Role of SETD2 in Clear Cell Renal Cell Carcinoma. Neoplasia.

[14] Li X., Song Y. (2021). Structure, function and inhibition of critical protein–protein interactions involving mixed lineage leukemia 1 and its fusion oncoproteins. J. Hematol. Oncol..

[15] de Rooij J.D., Hollink I.H., Arentsen-Peters S.T., van Galen J.F., Berna Beverloo H., Baruchel A., Trka J., Reinhardt D., Sonneveld E., Zimmermann M. (2013). NUP98/JARID1A is a novel recurrent abnormality in pediatric acute megakaryoblastic leukemia with a distinct HOX gene expression pattern. Leukemia.

[16] Ehrlich M. (2009). DNA hypomethylation in cancer cells. Epigenomics.

[17] Wong K.Y., So C.C., Loong F., Chung L.P., Lam W.W.L., Liang R., Li G.K.H., Jin N.-Y., Chim C. (2011). Epigenetic Inactivation of the miR-124-1 in Haematological Malignancies. PLoS ONE.

[18] Armas-Pineda C., Arenas-Huertero F., Pérezpeñia-Diazconti M., De León F.C.-P., Sosa-Sáinz G., Lezama P., Recillas-Targa F. (2007). Expression of PCAF, p300 and Gcn5 and more highly acetylated histone H4 in pediatric tumors. J. Exp. Clin. Cancer Res..

[19] Ramsawhook A., Lewis L., Coyle B., Ruzov A. (2017). Medulloblastoma and ependymoma cells display increased levels of 5-carboxylcytosine and elevated TET1 expression. Clin. Epigenet..

[20] Karlsson J., Valind A., Mengelbier L.H., Bredin S., Cornmark L., Jansson C., Wali A., Staaf J., Viklund B., Øra I. (2018). Four evolutionary trajectories underlie genetic intratumoral variation in childhood cancer. Nat. Genet..

[21] Haider Z., Landfors M., Golovleva I., Erlanson M., Schmiegelow K., Flægstad T., Kanerva J., Norén-Nyström U., Hultdin M., Degerman S. (2020). DNA methylation and copy number variation profiling of T-cell lymphoblastic leukemia and lymphoma. Blood Cancer J..

[22] Métais A., Bouchoucha Y., Kergrohen T., Dangouloff-Ros V., Maynadier X., Ajlil Y., Carton M., Yacoub W., Saffroy R., Figarella-Branger D. (2022). Pediatric spinal pilocytic astrocytomas form a distinct epigenetic subclass from pilocytic astrocytomas of other locations and diffuse leptomeningeal glioneuronal tumours. Acta Neuropathol..

[23] Zeng Q., Wang S., Tan J., Chen L., Wang J. (2021). The methylation level of TFAP2A is a potential diagnostic biomarker for retinoblastoma: An analytical validation study. Peerj.

[24] Sun R., Du C., Li J., Zhou Y., Xiong W., Xiang J., Liu J., Xiao Z., Fang L., Li Z. (2021). Systematic Investigation of DNA Methylation Associated with Platinum Chemotherapy Resistance Across 13 Cancer Types. Front. Pharmacol..

[25] Xu H., Li Y., Chen L., Wang C., Wang Q., Zhang H., Lin Y., Li Q., Pang T. (2015). SIRT2 mediates multidrug resistance in acute myelogenous leukemia cells via ERK1/2 signaling pathway. Int. J. Oncol..

[26] Zhao C., Yu H., Fan X., Niu W., Fan J., Sun S., Gong M., Zhao B., Fang Z., Chen X. (2022). GSK3β palmitoylation mediated by ZDHHC4 promotes tumorigenicity of glioblastoma stem cells in temozolomide-resistant glioblastoma through the EZH2–STAT3 axis. Oncogenesis.

[27] Pethusamy K., Seethy A., Dhar R., Karmakar A., Chaudhary S., Bakhshi S., P J.K., Chopra A., Chauhan S.S., Karmakar S. (2022). Loss of TET2 with reduced genomic 5-hmC is associated with adverse-risk AML. Leuk. Lymphoma.

[28] Shiba N., Ichikawa H., Taki T., Park M.-J., Jo A., Mitani S., Kobayashi T., Shimada A., Sotomatsu M., Arakawa H. (2013). *NUP98*-*NSD1* gene fusion and its related gene expression signature are strongly associated with a poor prognosis in pediatric acute myeloid leukemia. Genes Chromosom. Cancer.

[29] Schönung M., Meyer J., Nöllke P., Olshen A.B., Hartmann M., Murakami N., Wakamatsu M., Okuno Y., Plass C., Loh M.L. (2021). International Consensus Definition of DNA Methylation Subgroups in Juvenile Myelomonocytic Leukemia. Clin. Cancer Res..

[30] Sakhdari A., Class C., Montalban-Bravo G., Sasaki K., Bueso-Ramos C.E., Patel K.P., Routbort M.J., Loghavi S., Ok C.Y., Quesada A. (2022). Immunohistochemical loss of enhancer of Zeste Homolog 2 (EZH2) protein expression correlates with EZH2 alterations and portends a worse outcome in myelodysplastic syndromes. Mod. Pathol..

[31] Chen B.-Y., Song J., Hu C.-L., Chen S.-B., Zhang Q., Xu C.-H., Wu J.-C., Hou D., Sun M., Zhang Y.-L. (2020). SETD2 deficiency accelerates MDS-associated leukemogenesis via S100a9 in NHD13 mice and predicts poor prognosis in MDS. Blood.

[32] Bur H., Haapasaari K.-M., Turpeenniemi-Hujanen T., Kuittinen O., Auvinen P., Marin K., Soini Y., Karihtala P. (2017). Low Rap1-interacting factor 1 and sirtuin 6 expression predict poor outcome in radiotherapy-treated Hodgkin lymphoma patients. Leuk. Lymphoma.

[33] Kurimoto T., Kondo A., Ogino I., Fujimura J., Arakawa A., Arai H., Shimizu T. (2017). Effect of O6-methylguanine-DNA methyltransferase methylation in medulloblastoma. Mol. Clin. Oncol..

[34] Zhang F., Liu Y., Zhang Z., Li J., Wan Y., Zhang L., Wang Y., Li X., Xu Y., Fu X. (2016). 5-hydroxymethylcytosine loss is associated with poor prognosis for patients with WHO grade II diffuse astrocytomas. Sci. Rep..

[35] Li A.M., Dunham C., Tabori U., Carret A.-S., McNeely P.D., Johnston D., Lafay-Cousin L., Wilson B., Eisenstat D.D., Jabado N. (2015). EZH2 expression is a prognostic factor in childhood intracranial ependymoma: A Canadian Pediatric Brain Tumor Consortium study. Cancer.

[36] Applebaum M.A., Barr E.K., Karpus J., West-Szymanski D.C., Oliva M., Sokol E.A., Zhang S., Zhang Z., Zhang W., Chlenski A. (2020). 5-Hydroxymethylcytosine Profiles in Circulating Cell-Free DNA Associate with Disease Burden in Children with Neuroblastoma. Clin. Cancer Res..

[37] Qu Y., Liu L., Wang J., Xi W., Xia Y., Bai Q., Xiong Y., Long Q., Xu J., Guo J. (2016). Dot1l expression predicts adverse postoperative prognosis of patients with clear-cell renal cell carcinoma. Oncotarget.

[38] Bueno A.C., da Silva R.M.P., Stecchini M.F., Marrero-Gutiérrez J., Silva D.C.d.A.e., Cardinalli I., Scrideli C.A., Junqueira T., Molina C.A.F., Ramalho F.S. (2022). DNA methylation is a comprehensive marker for pediatric adrenocortical tumors. Endocr.-Relat. Cancer.

[39] Greenberg M.V.C., Bourc’His D. (2019). The diverse roles of DNA methylation in mammalian development and disease. Nat. Rev. Mol. Cell Biol..

[40] Lyko F. (2017). The DNA methyltransferase family: A versatile toolkit for epigenetic regulation. Nat. Rev. Genet..

[41] Short N.J., Dombret H., Adès L.M., Kantarjian H. (2022). The Evolution of Research and Therapy With Hypomethylating Agents in Acute Myeloid Leukemia and Myelodysplastic Syndrome: New Directions for Old Drugs. Cancer J..

[42] Ginder G.D., Williams D.C. (2017). Readers of DNA methylation, the MBD family as potential therapeutic targets. Pharmacol. Ther..

[43] Winters A.C., Maloney K.W., Treece A.L., Gore L., Franklin A.K. (2020). Single-center pediatric experience with venetoclax and azacitidine as treatment for myelodysplastic syndrome and acute myeloid leukemia. Pediatr. Blood Cancer.

[44] Gao J., Hu Y., Gao L., Xiao P., Lu J., Hu S. (2022). The effect of decitabine-combined minimally myelosuppressive regimen bridged allo-HSCT on the outcomes of pediatric MDS from 10 years’ experience of a single center. BMC Pediatr..

[45] Cheng S., Xiao P., Wang J., Li Z., Gao L., Zheng J., Hu Y., Ding X., Ling J., Lu Q. (2022). Decitabine combined with minimally myelosuppressive therapy for induction of remission in pediatric high-risk acute myeloid leukemia with chromosome 5q deletion: A report of three cases. Int. J. Hematol..

[46] Sun W., Triche T., Malvar J., Gaynon P., Sposto R., Yang X., Bittencourt H., Place A.E., Messinger Y., Fraser C. (2018). A phase 1 study of azacitidine combined with chemotherapy in childhood leukemia: A report from the TACL consortium. Blood.

[47] Hashmi S.K., Punia J.N., Marcogliese A.N., Gaikwad A.S., Fisher K.E., Roy A., Rao P., Lopez-Terrada D.H., Ringrose J., Loh M.L. (2019). Sustained remission with azacitidine monotherapy and an aberrant precursor B-lymphoblast population in juvenile myelomonocytic leukemia. Pediatr. Blood Cancer.

[48] Molina J.C., Asare J.M., Tuschong L., West R.R., Calvo K.R., Persky R., Boyce A.M., Hammoud D.A., Holland S.M., Hickstein D. (2020). Venetoclax/decitabine for a pediatric patient with chronic myelomonocytic leukemia. Pediatr. Blood Cancer.

[49] Hassler M.R., Klisaroska A., Kollmann K., Steiner I., Bilban M., Schiefer A.-I., Sexl V., Egger G. (2012). Antineoplastic activity of the DNA methyltransferase inhibitor 5-aza-2′-deoxycytidine in anaplastic large cell lymphoma. Biochimie.

[50] Arosio G., Sharma G.G., Villa M., Mauri M., Crespiatico I., Fontana D., Manfroni C., Mastini C., Zappa M., Magistroni V. (2021). Synergistic Drug Combinations Prevent Resistance in ALK+ Anaplastic Large Cell Lymphoma. Cancers.

[51] Tu Y., Xu L., Xu J., Bao Z., Tian W., Ye Y., Sun G., Miao Z., Chao H., You Y. (2022). Loss of deubiquitylase USP2 triggers development of glioblastoma via TGF-β signaling. Oncogene.

[52] Steinbügl M., Nemes K., Johann P., Kröncke T., Tüchert S., Gil da Costa M.J., Ebinger M., Schüller U., Sehested A., Hauser P. (2021). Clinical evidence for a biological effect of epigenetically active decitabine in relapsed or progressive rhabdoid tumors. Pediatr. Blood Cancer.

[53] Lin H.-Y., Chuang J.-H., Wang P.-W., Lin T.-K., Wu M.-T., Hsu W.-M., Chuang H.-C. (2020). 5-aza-2′-Deoxycytidine Induces a RIG-I-Related Innate Immune Response by Modulating Mitochondria Stress in Neuroblastoma. Cells.

[54] Krishnadas D.K., Bao L., Bai F., Chencheri S.C., Lucas K. (2014). Decitabine facilitates immune recognition of sarcoma cells by upregulating CT antigens, MHC molecules, and ICAM-1. Tumor Biol..

[55] Osuna M.A.L., Garcia-Lopez J., El Ayachi I., Fatima I., Khalid A.B., Kumpati J., Slayden A.V., Seagroves T.N., Miranda-Carboni G.A., Krum S.A. (2019). Activation of Estrogen Receptor Alpha by Decitabine Inhibits Osteosarcoma Growth and Metastasis. Cancer Res..

[56] Numoto K., Yoshida A., Sugihara S., Kunisada T., Morimoto Y., Yoneda Y., Fujita Y., Nishida K., Ouchida M., Ozaki T. (2009). Frequent methylation of RASSF1A in synovial sarcoma and the anti-tumor effects of 5-aza-2′-deoxycytidine against synovial sarcoma cell lines. J. Cancer Res. Clin. Oncol..

[57] Ueno-Yokohata H., Okita H., Nakasato K., Kiyokawa N. (2022). Hypermethylation of *RASSF1A* gene in pediatric rhabdoid tumor of the kidney and clear cell sarcoma of the kidney. Pediatr. Blood Cancer.

[58] Teng S., Ma C., Yu Y., Yi C. (2019). Hydroxyurea promotes TET1 expression and induces apoptosis in osteosarcoma cells. Biosci. Rep..

[59] Nakajima H., Kunimoto H. (2014). *TET2* as an epigenetic master regulator for normal and malignant hematopoiesis. Cancer Sci..

[60] Anderson N.M., Mucka P., Kern J.G., Feng H. (2017). The emerging role and targetability of the TCA cycle in cancer metabolism. Protein Cell.

[61] Wang Y., Shen N., Spurlin G., Korm S., Huang S., Anderson N.M., Huiting L.N., Liu H., Feng H. (2022). α-Ketoglutarate-Mediated DNA Demethylation Sustains T-Acute Lymphoblastic Leukemia upon TCA Cycle Targeting. Cancers.

[62] Carella A., Tejedor J.R., García M.G., Urdinguio R.G., Bayón G.F., Sierra M., Lopez V., García-Toraño E., Santamarina-Ojeda P., Pérez R.F. (2019). Epigenetic downregulation of TET3 reduces genome-wide 5hmC levels and promotes glioblastoma tumorigenesis. Int. J. Cancer.

[63] Kim H., Kang Y., Li Y., Chen L., Lin L., Johnson N.D., Zhu D., Robinson M.H., McSwain L., Barwick B.G. (2021). Ten-eleven translocation protein 1 modulates medulloblastoma progression. Genome Biol..

[64] Itoh H., Kadomatsu T., Tanoue H., Yugami M., Miyata K., Endo M., Morinaga J., Kobayashi E., Miyamoto T., Kurahashi R. (2018). TET2-dependent IL-6 induction mediated by the tumor microenvironment promotes tumor metastasis in osteosarcoma. Oncogene.

[65] Markouli M., Strepkos D., Piperi C. (2022). Impact of Histone Modifications and Their Therapeutic Targeting in Hematological Malignancies. Int. J. Mol. Sci..

[66] Zhao Z., Shilatifard A. (2019). Epigenetic modifications of histones in cancer. Genome Biol..

[67] Zhao S., Zhang X., Li H. (2018). Beyond histone acetylation—Writing and erasing histone acylations. Curr. Opin. Struct. Biol..

[68] Sun X.-J., Man N., Tan Y., Nimer S.D., Wang L. (2015). The Role of Histone Acetyltransferases in Normal and Malignant Hematopoiesis. Front. Oncol..

[69] Holmlund T., Lindberg M.J., Grander D., E Wallberg A. (2012). GCN5 acetylates and regulates the stability of the oncoprotein E2A-PBX1 in acute lymphoblastic leukemia. Leukemia.

[70] Kahl M., Brioli A., Bens M., Perner F., Kresinsky A., Schnetzke U., Hinze A., Sbirkov Y., Stengel S., Simonetti G. (2019). The acetyltransferase GCN5 maintains ATRA-resistance in non-APL AML. Leukemia.

[71] Valerio D.G., Xu H., Chen C.-W., Hoshii T., Eisold M.E., Delaney C., Cusan M., Deshpande A.J., Huang C.-H., Lujambio A. (2017). Histone Acetyltransferase Activity of MOF Is Required for *MLL-AF9* Leukemogenesis. Cancer Res..

[72] Man N., Mas G., Karl D.L., Sun J., Liu F., Yang Q., Torres-Martin M., Itonaga H., Martinez C., Chen S. (2021). p300 suppresses the transition of myelodysplastic syndromes to acute myeloid leukemia. JCI Insight.

[73] Farria A.T., Mustachio L.M., Akdemir Z.H.C., Dent S.Y. (2019). GCN5 HAT inhibition reduces human Burkitt lymphoma cell survival through reduction of MYC target gene expression and impeding BCR signaling pathways. Oncotarget.

[74] Gajer J.M., Furdas S.D., A Grunder A., Gothwal M., Heinicke U., Keller K.M., Colland F., Fulda S., Pahl H.L., Fichtner I. (2015). Histone acetyltransferase inhibitors block neuroblastoma cell growth in vivo. Oncogenesis.

[75] Bharathy N., Suriyamurthy S., Rao V.K., Ow J.R., Lim H.J., Chakraborty P., Vasudevan M., Dhamne C.A., Chang K.T.E., Min V.L.K. (2016). P/CAF mediates PAX3-FOXO1-dependent oncogenesis in alveolar rhabdomyosarcoma. J. Pathol..

[76] Gao Y.-Y., Ling Z.-Y., Zhu Y.-R., Shi C., Wang Y., Zhang X.-Y., Zhang Z.-Q., Jiang Q., Chen M.-B., Yang S. (2021). The histone acetyltransferase HBO1 functions as a novel oncogenic gene in osteosarcoma. Theranostics.

[77] Shi X., Fan M. (2019). Tip60-dependent acetylation of KDM2B promotes osteosarcoma carcinogenesis. J. Cell Mol. Med..

[78] Li G., Tian Y., Zhu W.-G. (2020). The Roles of Histone Deacetylases and Their Inhibitors in Cancer Therapy. Front. Cell Dev. Biol..

[79] McClure J.J., Li X., Chou C.J. (2018). Advances and Challenges of HDAC Inhibitors in Cancer Therapeutics. Adv. Cancer Res..

[80] Chen I.-C., Sethy B., Liou J.-P. (2020). Recent Update of HDAC Inhibitors in Lymphoma. Front. Cell Dev. Biol..

[81] Karol S.E., Do T.M.C., Mead P.E., Crews K., Panetta J.C., Alexander T., Taub J.W., Lacayo N.J., Heym K.M., Kuo D.J. (2020). Safety, pharmacokinetics, and pharmacodynamics of panobinostat in children, adolescents, and young adults with relapsed acute myeloid leukemia. Cancer.

[82] Pommert L., Schafer E.S., Malvar J., Gossai N., Florendo E., Pulakanti K., Heimbruch K., Stelloh C., Chi Y., Sposto R. (2022). Decitabine and vorinostat with FLAG chemotherapy in pediatric relapsed/refractory AML: Report from the therapeutic advances in childhood leukemia and lymphoma (TACL) consortium. Am. J. Hematol..

[83] Lillico R., Lawrence C.K., Lakowski T.M. (2018). Selective DOT1L, LSD1, and HDAC Class I Inhibitors Reduce HOXA9 Expression in MLL-AF9 Rearranged Leukemia Cells, But Dysregulate the Expression of Many Histone-Modifying Enzymes. J. Proteome Res..

[84] Zhao M., Duan Y., Wang J., Liu Y., Zhao Y., Wang H., Zhang L., Chen Z.-S., Hu Z., Wei L. (2022). Histone Deacetylase Inhibitor I3 Induces the Differentiation of Acute Myeloid Leukemia Cells with t (8; 21) or MLL Gene Translocation and Leukemic Stem-Like Cells. J. Oncol..

[85] Valiulienė G., Stirblytė I., Jasnauskaitė M., Borutinskaitė V., Navakauskienė R. (2017). Anti-leukemic effects of HDACi Belinostat and HMTi 3-Deazaneplanocin A on human acute promyelocytic leukemia cells. Eur. J. Pharmacol..

[86] Stumpel D.J.P.M., Schneider P., Seslija L., Osaki H., O Williams O., Pieters R., Stam R.W. (2011). Connectivity mapping identifies HDAC inhibitors for the treatment of t(4;11)-positive infant acute lymphoblastic leukemia. Leukemia.

[87] Oki Y., Copeland A., Younes A. (2011). Clinical development of panobinostat in classical Hodgkin’s lymphoma. Expert Rev. Hematol..

[88] Chu Y., Yahr A., Huang B., Ayello J., Barth M., Cairo M.S. (2017). Romidepsin alone or in combination with anti-CD20 chimeric antigen receptor expanded natural killer cells targeting Burkitt lymphoma in vitro and in immunodeficient mice. Oncoimmunology.

[89] Ma X.-J., Xu G., Li Z.-J., Chen F., Wu D., Miao J.-N., Zhan Y., Fan Y. (2019). HDAC-selective Inhibitor Cay10603 Has Single Anti-tumour Effect in Burkitt’s Lymphoma Cells by Impeding the Cell Cycle. Curr. Med. Sci..

[90] Perla A., Fratini L., Cardoso P.S., Nör C., Brunetto A.T., Brunetto A.L., De Farias C.B., Jaeger M., Roesler R. (2020). Histone Deacetylase Inhibitors in Pediatric Brain Cancers: Biological Activities and Therapeutic Potential. Front. Cell Dev. Biol..

[91] Nawar N., Bukhari S., Adile A.A., Suk Y., Manaswiyoungkul P., Toutah K., Olaoye O.O., Raouf Y.S., Sedighi A., Garcha H.K. (2022). Discovery of HDAC6-Selective Inhibitor NN-390 with in Vitro Efficacy in Group 3 Medulloblastoma. J. Med. Chem..

[92] Was H., Krol S.K., Rotili D., Mai A., Wojtas B., Kaminska B., Maleszewska M. (2019). Histone deacetylase inhibitors exert anti-tumor effects on human adherent and stem-like glioma cells. Clin. Epigenet..

[93] Su J.M., Kilburn L.B., Mansur D.B., Krailo M., Buxton A., Adekunle A., Gajjar A., Adamson P.C., Weigel B., Fox E. (2021). Phase I/II trial of vorinostat and radiation and maintenance vorinostat in children with diffuse intrinsic pontine glioma: A Children’s Oncology Group report. Neuro-Oncol..

[94] Milde T., Kleber S., Korshunov A., Witt H., Hielscher T., Koch P., Kopp H.-G., Jugold M., Deubzer H.E., Oehme I. (2011). A novel human high-risk ependymoma stem cell model reveals the differentiation-inducing potential of the histone deacetylase inhibitor Vorinostat. Acta Neuropathol..

[95] Kaneda D., Iehara T., Kikuchi K., Sugimoto Y., Nakagawa N., Yagyu S., Miyachi M., Konishi E., Sakai T., Hosoi H. (2022). The histone deacetylase inhibitor OBP-801 has in vitro/in vivo anti-neuroblastoma activity. Pediatr. Int..

[96] Panicker J., Li Z., McMahon C., Sizer C., Steadman K., Piekarz R., Bates S.E., Thiele C.J. (2010). Romidepsin (FK228/depsipeptide) controls growth and induces apoptosis in neuroblastoma tumor cells. Cell Cycle.

[97] Lu X., Liu M., Yang J., Que Y., Zhang X. (2022). Panobinostat enhances NK cell cytotoxicity in soft tissue sarcoma. Clin. Exp. Immunol..

[98] Pham T.Q., Robinson K., Xu L., Pavlova M.N., Skapek S.X., Chen E.Y. (2020). HDAC6 promotes growth, migration/invasion, and self-renewal of rhabdomyosarcoma. Oncogene.

[99] García-Domínguez D.J., Hajji N., Sánchez-Molina S., Figuerola-Bou E., de Pablos R.M., Espinosa-Oliva A.M., Andrés-León E., Terrón-Camero L.C., Flores-Campos R., Pascual-Pasto G. (2021). Selective inhibition of HDAC6 regulates expression of the oncogenic driver EWSR1-FLI1 through the EWSR1 promoter in Ewing sarcoma. Oncogene.

[100] Yan-Fang T., Zhi-Heng L., Li-Xiao X., Fang F., Jun L., Gang L., Lan C., Na-Na W., Xiao-Juan D., Li-Chao S. (2015). Molecular Mechanism of the Cell Death Induced by the Histone Deacetylase Pan Inhibitor LBH589 (Panobinostat) in Wilms Tumor Cells. PLoS ONE.

[101] Rivas M., Johnston M.E., Gulati R., Kumbaji M., Aguiar T.F.M., Timchenko L., Krepischi A., Shin S., Bondoc A., Tiao G. (2021). HDAC1-Dependent Repression of Markers of Hepatocytes and P21 Is Involved in Development of Pediatric Liver Cancer. Cell. Mol. Gastroenterol. Hepatol..

[102] Kaczmarek J.V., Bogan C.M., Pierce J.M., Tao Y.K., Chen S.-C., Liu Q., Liu X., Boyd K.L., Calcutt M.W., Bridges T.M. (2021). Intravitreal HDAC Inhibitor Belinostat Effectively Eradicates Vitreous Seeds Without Retinal Toxicity In Vivo in a Rabbit Retinoblastoma Model. Investig. Opthalmology Vis. Sci..

[103] Huegel J., Dinh C.T., Martinelli M., Bracho O., Rosario R., Hardin H., Estivill M., Griswold A., Gultekin S., Liu X.-Z. (2022). CUDC907, a dual phosphoinositide-3 kinase/histone deacetylase inhibitor, promotes apoptosis of NF2 Schwannoma cells. Oncotarget.

[104] Gaál Z., Csernoch L. (2020). Impact of Sirtuin Enzymes on the Altered Metabolic Phenotype of Malignantly Transformed Cells. Front. Oncol..

[105] Chalkiadaki A., Guarente L. (2015). The multifaceted functions of sirtuins in cancer. Nat. Rev. Cancer.

[106] Jin Y., Cao Q., Chen C., Du X., Jin B., Pan J. (2015). Tenovin-6-mediated inhibition of SIRT1/2 induces apoptosis in acute lymphoblastic leukemia (ALL) cells and eliminates ALL stem/progenitor cells. BMC Cancer.

[107] Wang F., Li Z., Zhou J., Wang G., Zhang W., Xu J., Liang A. (2021). SIRT1 regulates the phosphorylation and degradation of P27 by deacetylating CDK2 to promote T-cell acute lymphoblastic leukemia progression. J. Exp. Clin. Cancer Res..

[108] Okasha S.M., Itoh M., Tohda S. (2020). Sirtuin 1 Activation Suppresses the Growth of T-lymphoblastic Leukemia Cells by Inhibiting NOTCH and NF-κB Pathways. Anticancer Res..

[109] Zhou L., Wang Q., Chen X., Fu L., Zhang X., Wang L., Deng A., Li D., Liu J., Lv N. (2016). AML1–ETO promotes SIRT1 expression to enhance leukemogenesis of t(8;21) acute myeloid leukemia. Exp. Hematol..

[110] Yan D., Franzini A., Pomicter A.D., Halverson B.J., Antelope O., Mason C.C., Ahmann J.M., Senina A.V., Vellore N.A., Jones C.L. (2021). SIRT5 Is a Druggable Metabolic Vulnerability in Acute Myeloid Leukemia. Blood Cancer Discov..

[111] Sun J., He X., Zhu Y., Ding Z., Dong H., Feng Y., Du J., Wang H., Wu X., Zhang L. (2018). SIRT1 Activation Disrupts Maintenance of Myelodysplastic Syndrome Stem and Progenitor Cells by Restoring TET2 Function. Cell Stem Cell.

[112] Vettraino M., Manerba M., Govoni M., Di Stefano G. (2013). Galloflavin suppresses lactate dehydrogenase activity and causes MYC downregulation in Burkitt lymphoma cells through NAD/NADH-dependent inhibition of sirtuin-1. Anti-Cancer Drugs.

[113] Miller J.J., Fink A., A Banagis J., Nagashima H., Subramanian M., Lee C.K., Melamed L., Tummala S.S., Tateishi K., Wakimoto H. (2020). Sirtuin activation targets IDH-mutant tumors. Neuro-Oncol..

[114] Wang T., Li X., Sun S.-L. (2020). EX527, a Sirt-1 inhibitor, induces apoptosis in glioma via activating the p53 signaling pathway. Anti-Cancer Drugs.

[115] Mu P., Liu K., Lin Q., Yang W., Liu D., Lin Z., Shao W., Ji T. (2018). Sirtuin 7 promotes glioma proliferation and invasion through activation of the ERK/STAT3 signaling pathway. Oncol. Lett..

[116] Shi J., Sun S., Xing S., Huang C., Huang Y., Wang Q., Xue X., Chen Z., Wang Y., Huang Z. (2022). Fraxinellone inhibits progression of glioblastoma via regulating the SIRT3 signaling pathway. Biomed. Pharmacother..

[117] Dou F., Tian Z., Yang X., Li J., Wang R., Gao J. (2022). Valemetostat: First approval as a dual inhibitor of EZH1/2 to treat adult T-cell leukemia/lymphoma. Drug Discov. Ther..

[118] Song H.Y., Rellinger E.J., Park S.-H., Paul P., Qiao J., Vasilopoulos A., Özden O., Gius D., Chung D.H. (2018). Inhibition of Sirtuin 6 Induces Neuroblastoma Differentiation. Anticancer Res..

[119] Ma L., Maruwge W., Strambi A., D’Arcy P., Pellegrini P., Kis L., De Milito A., Lain S., Brodin B. (2014). SIRT1 and SIRT2 inhibition impairs pediatric soft tissue sarcoma growth. Cell Death Dis..

[120] Ban J., Aryee D.N., Fourtouna A., van der Ent W., Kauer M., Niedan S., Machado I., Rodriguez-Galindo C., Tirado O.M., Schwentner R. (2014). Suppression of Deacetylase SIRT1 Mediates Tumor-Suppressive NOTCH Response and Offers a Novel Treatment Option in Metastatic Ewing Sarcoma. Cancer Res..

[121] Wang C., Piao C., Liu J., Zhang Z., Zhu Y., Kong C. (2020). Mammalian SIRT4 is a tumor suppressor of clear cell renal cell carcinoma by inhibiting cancer proliferation, migration and invasion. Cancer Biomarkers.

[122] An J., Yang J., Yao Y., Lu K., Zhao Z., Yu M., Zhu Y. (2021). Sirtuin 6 regulates the proliferation and survival of clear cell renal cell carcinoma cells via B-cell lymphoma 2. Oncol. Lett..

[123] Ouyang L., Yi L., Li J., Yi S., Li S., Liu P., Yang X. (2018). SIRT6 overexpression induces apoptosis of nasopharyngeal carcinoma by inhibiting NF-κB signaling. OncoTargets Ther..

[124] Greer E.L., Shi Y. (2012). Histone methylation: A dynamic mark in health, disease and inheritance. Nat. Rev. Genet..

[125] Milosevich N., Hof F. (2015). Chemical Inhibitors of Epigenetic Methyllysine Reader Proteins. Biochemistry.

[126] D’Angelo V., Iannotta A., Ramaglia M., Lombardi A., Zarone M.R., Desiderio V., Affinita M.C., Pecoraro G., Di Martino M., Indolfi P. (2015). EZH2 is increased in paediatric T-cell acute lymphoblastic leukemia and is a suitable molecular target in combination treatment approaches. J. Exp. Clin. Cancer Res..

[127] Ito J., Yamagata K., Shinohara H., Shima Y., Katsumoto T., Aikawa Y., Kitabayashi I. (2022). Dual inhibition of EZH1/2 induces cell cycle arrest of B cell acute lymphoblastic leukemia cells through upregulation of CDKN1C and TP53INP1. Int. J. Hematol..

[128] Al-Ghabkari A., Narendran A. (2021). Targeting EZH2-mediated methylation of histone 3 inhibits proliferation of pediatric acute monocytic leukemia cells in vitro. Cancer Biol. Ther..

[129] Zheng Z., Li L., Li G., Zhang Y., Dong C., Ren F., Chen W., Ma Y. (2021). EZH2/EHMT2 Histone Methyltransferases Inhibit the Transcription of DLX5 and Promote the Transformation of Myelodysplastic Syndrome to Acute Myeloid Leukemia. Front. Cell Dev. Biol..

[130] Smith J.J., Porter-Scott M., Chesworth R., Moyer M.P., Copeland R.A., Richon V.M., Uenaka T., Pollock R.M., Kuntz K.W., Yokoi A. (2014). Selective inhibition of EZH2 by EPZ-6438 leads to potent antitumor activity in EZH2-mutant non-Hodgkin lymphoma. Mol. Cancer Ther..

[131] Italiano A., Soria J.C., Toulmonde M., Michot J.M., Lucchesi C., Varga A., Coindre J.M., Blakemore S.J., Clawson A., Suttle B. (2018). Tazemetostat, an EZH2 inhibitor, in relapsed or refractory B-cell non-Hodgkin lymphoma and advanced solid tumours: A first-in-human, open-label, phase 1 study. Lancet Oncol..

[132] Miele E., Valente S., Alfano V., Silvano M., Mellini P., Borovika D., Marrocco B., Po A., Besharat Z.M., Catanzaro G. (2017). The histone methyltransferase EZH2 as a druggable target in SHH medulloblastoma cancer stem cells. Oncotarget.

[133] Mohammad F., Weissmann S., Leblanc B., Pandey D.P., Hojfeldt J., Comet I., Zheng C., Johansen J.V., Rapin N., Porse N.R.B.T. (2017). EZH2 is a potential therapeutic target for H3K27M-mutant pediatric gliomas. Nat. Med..

[134] Bownes L.V., Williams A.P., Marayati R., Stafman L.L., Markert H., Quinn C.H., Wadhwani N., Aye J.M., Stewart J.E., Yoon K.J. (2021). EZH2 inhibition decreases neuroblastoma proliferation and in vivo tumor growth. PLoS ONE.

[135] Schmidt A., Behrendt L., Eybe J., Warmann S.W., Schleicher S., Fuchs J., Schmid E. (2021). The Effect of Direct and Indirect EZH2 Inhibition in Rhabdomyosarcoma Cell Lines. Cancers.

[136] Simeone N., Frezza A.M., Zaffaroni N., Stacchiotti S. (2021). Tazemetostat for advanced epithelioid sarcoma: Current status and future perspectives. Future Oncol..

[137] Shinohara H., Sawado R., Nakagawa M., Hattori A., Yamagata K., Tauchi K., Ito J., Kuwahara Y., Okuda T., Ogawa C. (2022). Dual targeting of EZH1 and EZH2 for the treatment of malignant rhabdoid tumors. Mol. Ther. -Oncolytics.

[138] Huang H., Howard C.A., Zari S., Cho H.J., Shukla S., Li H., Ndoj J., González-Alonso P., Nikolaidis C., Abbott J. (2020). Covalent inhibition of NSD1 histone methyltransferase. Nat. Chem. Biol..

[139] Madrazo E., Ruano D., Abad L., Alonso-Gómez E., Sánchez-Valdepeñas C., González-Murillo Á., Ramírez M., Redondo-Muñoz J. (2018). G9a Correlates with VLA-4 Integrin and Influences the Migration of Childhood Acute Lymphoblastic Leukemia Cells. Cancers.

[140] José-Enériz E.S., Agirre X., Rabal O., Vilas-Zornoza A., Sanchez-Arias J.A., Miranda E., Ugarte A., Roa S., Paiva B., de Mendoza A.E.-H. (2017). Discovery of first-in-class reversible dual small molecule inhibitors against G9a and DNMTs in hematological malignancies. Nat. Commun..

[141] Klonou A., Korkolopoulou P., Gargalionis A.N., Kanakoglou D.S., Katifelis H., Gazouli M., Chlamydas S., Mitsios A., Kalamatianos T., Stranjalis G. (2021). Histone Mark Profiling in Pediatric Astrocytomas Reveals Prognostic Significance of H3K9 Trimethylation and Histone Methyltransferase SUV39H1. Neurotherapeutics.

[142] Siddaway R., Canty L., Pajovic S., Milos S., Coyaud E., Sbergio S.-G., Anguraj A.K.V., Lubanszky E., Yun H.Y., Portante A. (2022). Oncohistone interactome profiling uncovers contrasting oncogenic mechanisms and identifies potential therapeutic targets in high grade glioma. Acta Neuropathol..

[143] Ke X.-X., Zhang D., Zhu S., Xia Q., Xiang Z., Cui H. (2019). Inhibition of H3K9 Methyltransferase G9a Repressed Cell Proliferation and Induced Autophagy in Neuroblastoma Cells. PLoS ONE.

[144] Pal A., Leung J.Y., Ang G.C.K., Rao V.K., Pignata L., Lim H.J., Hebrard M., Chang K.T., Lee V.K., Guccione E. (2020). EHMT2 epigenetically suppresses Wnt signaling and is a potential target in embryonal rhabdomyosarcoma. eLife.

[145] García-Domínguez D.J., Hajji N., López-Alemany R., Sánchez-Molina S., Figuerola-Bou E., Civanto F.J.M., Rello-Varona S., Andrés-León E., Benito A., Keun H.C. (2022). Selective histone methyltransferase G9a inhibition reduces metastatic development of Ewing sarcoma through the epigenetic regulation of NEU1. Oncogene.

[146] Wang J., Yin X., He W., Xue W., Zhang J., Huang Y. (2020). SUV39H1 deficiency suppresses clear cell renal cell carcinoma growth by inducing ferroptosis. Acta Pharm. Sin. B.

[147] Min J., Feng Q., Li Z., Zhang Y., Xu R.-M. (2003). Structure of the Catalytic Domain of Human DOT1L, a Non-SET Domain Nucleosomal Histone Methyltransferase. Cell.

[148] Bernt K.M., Zhu N., Sinha A.U., Vempati S., Faber J., Krivtsov A.V., Feng Z., Punt N., Daigle A., Bullinger L. (2011). MLL-Rearranged Leukemia Is Dependent on Aberrant H3K79 Methylation by DOT1L. Cancer Cell.

[149] Brzezinka K., Nevedomskaya E., Lesche R., Steckel M., Eheim A.L., Haegebarth A., Stresemann C. (2019). Functional diversity of inhibitors tackling the differentiation blockage of MLL-rearranged leukemia. J. Hematol. Oncol..

[150] Yi Y., Ge S. (2022). Targeting the histone H3 lysine 79 methyltransferase DOT1L in MLL-rearranged leukemias. J. Hematol. Oncol..

[151] Rau R.E., Rodriguez B.A., Luo M., Jeong M., Rosen A., Rogers J.H., Campbell C.T., Daigle S.R., Deng L., Song Y. (2016). DOT1L as a therapeutic target for the treatment of DNMT3A-mutant acute myeloid leukemia. Blood.

[152] Wong M., Tee A.E., Milazzo G., Bell J.L., Poulos R.C., Atmadibrata B., Sun Y., Jing D., Ho N., Ling D. (2017). The Histone Methyltransferase DOT1L Promotes Neuroblastoma by Regulating Gene Transcription. Cancer Res..

[153] Mao Y., Sun Y., Wu Z., Zheng J., Zhang J., Zeng J., Lee C., Kim J.K. (2021). Targeting of histone methyltransferase DOT1L plays a dual role in chemosensitization of retinoblastoma cells and enhances the efficacy of chemotherapy. Cell Death Dis..

[154] Smith E., Zhou W., Shindiapina P., Sif S., Li C., Baiocchi R.A. (2018). Recent advances in targeting protein arginine methyltransferase enzymes in cancer therapy. Expert Opin. Ther. Targets.

[155] He X., Zhu Y., Lin Y.-C., Li M., Du J., Dong H., Sun J., Zhu L., Wang H., Ding Z. (2019). PRMT1-mediated FLT3 arginine methylation promotes maintenance of FLT3-ITD+ acute myeloid leukemia. Blood.

[156] Zhu Y., He X., Lin Y.-C., Dong H., Zhang L., Chen X., Wang Z., Shen Y., Li M., Wang H. (2019). Targeting PRMT1-mediated FLT3 methylation disrupts maintenance of MLL-rearranged acute lymphoblastic leukemia. Blood.

[157] Fong J.Y., Pignata L., Goy P.-A., Kawabata K.C., Lee S.C.-W., Koh C.M., Musiani D., Massignani E., Kotini A.G., Penson A. (2019). Therapeutic Targeting of RNA Splicing Catalysis through Inhibition of Protein Arginine Methylation. Cancer Cell.

[158] Wang C., Jiang H., Jin J., Xie Y., Chen Z., Zhang H., Lian F., Liu Y.-C., Zhang C., Ding H. (2017). Development of Potent Type I Protein Arginine Methyltransferase (PRMT) Inhibitors of Leukemia Cell Proliferation. J. Med. Chem..

[159] Chung J., Karkhanis V., Baiocchi R.A., Sif S. (2019). Protein arginine methyltransferase 5 (PRMT5) promotes survival of lymphoma cells via activation of WNT/β-catenin and AKT/GSK3β proliferative signaling. J. Biol. Chem..

[160] Gu X., He M., Lebedev T., Lin C.-H., Hua Z.-Y., Zheng Y.G., Li Z.-J., Yang J.-Y., Li X.-G. (2022). PRMT1 is an important factor for medulloblastoma cell proliferation and survival. Biochem. Biophys. Rep..

[161] Wynn D.T., Rodriguez-Blanco J., Long J., Yang F., Shen C., Fei D., Tang H.-Y., Hanson D., Robbins D.J. (2022). PROTEIN ARGININE METHYLTRANSFERASE 5 regulates SHH-subgroup medulloblastoma progression. Neuro-Oncol. Adv..

[162] Janisiak J., Kopytko P., Tkacz M., Rogińska D., Perużyńska M., Machaliński B., Pawlik A., Tarnowski M. (2021). Protein Arginine Methyltransferase (PRMT) Inhibitors—AMI-1 and SAH Are Effective in Attenuating Rhabdomyosarcoma Growth and Proliferation in Cell Cultures. Int. J. Mol. Sci..

[163] Shi Y., Whetstine J.R. (2007). Dynamic Regulation of Histone Lysine Methylation by Demethylases. Mol. Cell.

[164] Cuthbert G.L., Daujat S., Snowden A.W., Erdjument-Bromage H., Hagiwara T., Yamada M., Schneider R., Gregory P.D., Tempst P., Bannister A.J. (2004). Histone Deimination Antagonizes Arginine Methylation. Cell.

[165] Kohrogi K., Hino S., Sakamoto A., Anan K., Takase R., Araki H., Hino Y., Araki K., Sato T., Nakamura K. (2021). LSD1 defines erythroleukemia metabolism by controlling the lineage-specific transcription factors GATA1 and C/EBPα. Blood Adv..

[166] Yang C., Fang Y., Luo X., Teng D., Liu Z., Zhou Y., Liao G. (2022). Discovery of natural product-like spirooxindole derivatives as highly potent and selective LSD1/KDM1A inhibitors for AML treatment. Bioorganic Chem..

[167] Saito S., Kikuchi J., Koyama D., Sato S., Koyama H., Osada N., Kuroda Y., Akahane K., Inukai T., Umehara T. (2019). Eradication of Central Nervous System Leukemia of T-Cell Origin with a Brain-Permeable LSD1 Inhibitor. Clin. Cancer Res..

[168] Theisen E.R., Pishas K.I., Saund R.S., Lessnick S.L. (2016). Therapeutic opportunities in Ewing sarcoma: EWS-FLI inhibition via LSD1 targeting. Oncotarget.

[169] Jiang A., Wu W., Xu C., Mao L., Ao S., Guo H., Sun X., Tao J., Sang Y., Huang G. (2022). SP2509, a Selective Inhibitor of LSD1, Suppresses Retinoblastoma Growth by Downregulating β-catenin Signaling. Investig. Opthalmology Vis. Sci..

[170] Li Y., Wang C., Gao H., Gu J., Zhang Y., Zhang Y., Xie M., Cheng X., Yang M., Zhang W. (2022). KDM4 inhibitor SD49-7 attenuates leukemia stem cell via KDM4A/MDM2/p21^CIP1^ axis. Theranostics.

[171] Li Y., Zhang M., Sheng M., Zhang P., Chen Z., Xing W., Bai J., Cheng T., Yang F.-C., Zhou Y. (2018). Therapeutic potential of GSK-J4, a histone demethylase KDM6B/JMJD3 inhibitor, for acute myeloid leukemia. J. Cancer Res. Clin. Oncol..

[172] Illiano M., Conte M., Salzillo A., Ragone A., Spina A., Nebbioso A., Altucci L., Sapio L., Naviglio S. (2020). The KDM Inhibitor GSKJ4 Triggers CREB Downregulation via a Protein Kinase A and Proteasome-Dependent Mechanism in Human Acute Myeloid Leukemia Cells. Front. Oncol..

[173] Qi D., Wang J., Zhao Y., Yang Y., Wang Y., Wang H., Wang L., Wang Z., Xu X., Hu Z. (2022). JMJD1C-regulated lipid synthesis contributes to the maintenance of *MLL*-rearranged acute myeloid leukemia. Leuk. Lymphoma.

[174] Xu S., Wang S., Xing S., Yu D., Rong B., Gao H., Sheng M., Tan Y., Zhang Y., Sun X. (2021). KDM5A suppresses PML-RARα target gene expression and APL differentiation through repressing H3K4me2. Blood Adv..

[175] Singh S., Abu-Zaid A., Jin H., Fang J., Wu Q., Wang T., Feng H., Quarni W., Shao Y., Maxham L. (2022). Targeting KDM4 for treating PAX3-FOXO1–driven alveolar rhabdomyosarcoma. Sci. Transl. Med..

[176] Sobral L.M., Sechler M., Parrish J.K., McCann T.S., Jones K.L., Black J.C., Jedlicka P. (2020). KDM3A/Ets1/MCAM axis promotes growth and metastatic properties in Rhabdomyosarcoma. Genes Cancer.

[177] Chen B., Chen H., Lu S., Zhu X., Que Y., Zhang Y., Huang J., Zhang L., Sun F., Wang J. (2022). KDM5B promotes tumorigenesis of Ewing sarcoma via FBXW7/CCNE1 axis. Cell Death Dis..

[178] Wang W., Bin Wang B. (2022). KDM3A-mediated SP1 activates PFKFB4 transcription to promote aerobic glycolysis in osteosarcoma and augment tumor development. BMC Cancer.

[179] Zhou J., Simon J.M., Liao C., Zhang C., Hu L., Zurlo G., Liu X., Fan C., Hepperla A., Jia L. (2022). An oncogenic JMJD6-DGAT1 axis tunes the epigenetic regulation of lipid droplet formation in clear cell renal cell carcinoma. Mol. Cell.

[180] Romero O.A., Vilarrubi A., Alburquerque-Bejar J.J., Gomez A., Andrades A., Trastulli D., Pros E., Setien F., Verdura S., Farré L. (2021). SMARCA4 deficient tumours are vulnerable to KDM6A/UTX and KDM6B/JMJD3 blockade. Nat. Commun..

[181] Du Q., Luu P.-L., Stirzaker C., Clark S.J. (2015). Methyl-CpG-binding domain proteins: Readers of the epigenome. Epigenomics.

[182] Mahmood N., Rabbani S.A. (2019). DNA Methylation Readers and Cancer: Mechanistic and Therapeutic Applications. Front. Oncol..

[183] Zhou M., Zhou K., Cheng L., Chen X., Wang J., Wang X.-M., Zhang Y., Yu Q., Zhang S., Wang D. (2018). MBD2 Ablation Impairs Lymphopoiesis and Impedes Progression and Maintenance of T-ALL. Cancer Res..

[184] Park S., Sater A.H.A., Fahrmann J.F., Irajizad E., Cai Y., Katayama H., Vykoukal J., Kobayashi M., Dennison J.B., Garcia-Manero G. (2022). Novel UHRF1-MYC Axis in Acute Lymphoblastic Leukemia. Cancers.

[185] Sharma K., Singh J., Frost E.E., Pillai P.P. (2018). MeCP2 overexpression inhibits proliferation, migration and invasion of C6 glioma by modulating ERK signaling and gene expression. Neurosci. Lett..

[186] Zhu D., Hunter S.B., Vertino P.M., Van Meir E.G. (2011). Overexpression of MBD2 in Glioblastoma Maintains Epigenetic Silencing and Inhibits the Antiangiogenic Function of the Tumor Suppressor Gene *BAI1*. Cancer Res..

[187] Gong L.-J., Wang X.-Y., Yao X.-D., Wu X., Gu W.-Y. (2021). CircESRP1 inhibits clear cell renal cell carcinoma progression through the CTCF-mediated positive feedback loop. Cell Death Dis..

[188] Filippakopoulos P., Picaud S., Mangos M., Keates T., Lambert J.-P., Barsyte-Lovejoy D., Felletar I., Volkmer R., Müller S., Pawson T. (2012). Histone Recognition and Large-Scale Structural Analysis of the Human Bromodomain Family. Cell.

[189] Ali I., Conrad R.J., Verdin E., Ott M. (2018). Lysine Acetylation Goes Global: From Epigenetics to Metabolism and Therapeutics. Chem. Rev..

[190] Sanchez R., Zhou M.-M. (2009). The role of human bromodomains in chromatin biology and gene transcription. Curr. Opin. Drug Discov. Dev..

[191] Li J., Galbo P.M., Gong W., Storey A.J., Tsai Y.-H., Yu X., Ahn J.H., Guo Y., Mackintosh S.G., Edmondson R.D. (2021). ZMYND11-MBTD1 induces leukemogenesis through hijacking NuA4/TIP60 acetyltransferase complex and a PWWP-mediated chromatin association mechanism. Nat. Commun..

[192] Liu Y., Li Q., Alikarami F., Barrett D.R., Mahdavi L., Li H., Tang S., Khan T.A., Michino M., Hill C. (2022). Small-Molecule Inhibition of the Acyl-Lysine Reader ENL as a Strategy against Acute Myeloid Leukemia. Cancer Discov..

[193] Baell J.B., Leaver D.J., Hermans S.J., Kelly G.L., Brennan M.S., Downer N.L., Nguyen N., Wichmann J., McRae H.M., Yang Y. (2018). Inhibitors of histone acetyltransferases KAT6A/B induce senescence and arrest tumour growth. Nature.

[194] Boi M., Todaro M., Vurchio V., Yang S.N., Moon J., Kwee I., Rinaldi A., Pan H., Crescenzo R., Cheng M. (2016). Therapeutic efficacy of the bromodomain inhibitor OTX015/MK-8628 in ALK-positive anaplastic large cell lymphoma: An alternative modality to overcome resistant phenotypes. Oncotarget.

[195] Bandopadhayay P., Bergthold G., Nguyen B., Schubert S., Gholamin S., Tang Y., Bolin S., Schumacher S.E., Zeid R., Masoud S. (2014). BET Bromodomain Inhibition of *MYC*-Amplified Medulloblastoma. Clin. Cancer Res..

[196] Berenguer-Daizé C., Astorgues-Xerri L., Odore E., Cayol M., Cvitkovic E., Noel K., Bekradda M., MacKenzie S., Rezai K., Lokiec F. (2016). OTX015 (MK-8628), a novel BET inhibitor, displays in vitro and in vivo antitum or effects alone and in combination with conventional therapies in glioblastoma models. Int. J. Cancer.

[197] Ishi Y., Zhang Y., Zhang A., Sasaki T., Piunti A., Suri A., Watanabe J., Abe K., He X., Katagi H. (2022). Therapeutic Targeting of EZH2 and BET BRD4 in Pediatric Rhabdoid Tumors. Mol. Cancer Ther..

[198] Servidei T., Meco D., Martini M., Battaglia A., Granitto A., Buzzonetti A., Babini G., Massimi L., Tamburrini G., Scambia G. (2021). The BET Inhibitor OTX015 Exhibits In Vitro and In Vivo Antitumor Activity in Pediatric Ependymoma Stem Cell Models. Int. J. Mol. Sci..

[199] Maurer-Stroh S., Dickens N.J., Hughes-Davies L., Kouzarides T., Eisenhaber F., Ponting C.P. (2003). The Tudor domain ‘Royal Family’: Tudor, plant Agenet, Chromo, PWWP and MBT domains. Trends Biochem. Sci..

[200] Wang S., Denton K.E., Hobbs K.F., Weaver T., McFarlane J.M.B., Connelly K.E., Gignac M.C., Milosevich N., Hof F., Paci I. (2019). Optimization of Ligands Using Focused DNA-Encoded Libraries To Develop a Selective, Cell-Permeable CBX8 Chromodomain Inhibitor. ACS Chem. Biol..

[201] Liu L., Guo X., Wang Y., Li G., Yu Y., Song Y., Zeng C., Ding Z., Qiu Y., Yan F. (2023). Loss of Wdr5 attenuates MLL-rearranged leukemogenesis by suppressing Myc targets. Biochim. Biophys. Acta Mol. Basis Dis..

[202] Tsai H.-I., Wu Y., Huang R., Su D., Wu Y., Liu X., Wang L., Xu Z., Pang Y., Sun C. (2021). PHF6 functions as a tumor suppressor by recruiting methyltransferase SUV39H1 to nucleolar region and offers a novel therapeutic target for PHF6-muntant leukemia. Acta Pharm. Sin. B.

[203] Azagra A., Cobaleda C. (2022). NSD2 as a Promising Target in Hematological Disorders. Int. J. Mol. Sci..

[204] Bao Z., Xu X., Liu Y., Chao H., Lin C., Li Z., You Y., Liu N., Ji J. (2017). CBX7 negatively regulates migration and invasion in glioma via Wnt/β-catenin pathway inactivation. Oncotarget.

[205] Helming K.C., Wang X., Roberts C.W. (2014). Vulnerabilities of Mutant SWI/SNF Complexes in Cancer. Cancer Cell.

[206] Hasan N., Ahuja N. (2019). The Emerging Roles of ATP-Dependent Chromatin Remodeling Complexes in Pancreatic Cancer. Cancers.

[207] Clapier C.R., Iwasa J., Cairns B.R., Peterson C.L. (2017). Mechanisms of action and regulation of ATP-dependent chromatin-remodelling complexes. Nat. Rev. Mol. Cell Biol..

[208] Rago F., Rodrigues L.U., Bonney M., Sprouffske K., Kurth E., Elliott G., Ambrose J., Aspesi P., Oborski J., Chen J.T. (2022). Exquisite Sensitivity to Dual BRG1/BRM ATPase Inhibitors Reveals Broad SWI/SNF Dependencies in Acute Myeloid Leukemia. Mol. Cancer Res..

[209] Zikmund T., Paszekova H., Kokavec J., Kerbs P., Thakur S., Turkova T., Tauchmanova P., Greif P.A., Stopka T. (2020). Loss of ISWI ATPase SMARCA5 (SNF2H) in Acute Myeloid Leukemia Cells Inhibits Proliferation and Chromatid Cohesion. Int. J. Mol. Sci..

[210] Panditharatna E., Marques J.G., Wang T., Trissal M.C., Liu I., Jiang L., Beck A., Groves A., Dharia N.V., Li D. (2022). BAF complex maintains glioma stem cells in pediatric H3K27M-glioma. Cancer Discov..

[211] Machado R.A.C., Schneider H., DeOcesano-Pereira C., Lichtenstein F., Andrade F., Fujita A., Trombetta-Lima M., Weller M., Bowman-Colin C., Sogayar M.C. (2019). CHD7 promotes glioblastoma cell motility and invasiveness through transcriptional modulation of an invasion signature. Sci. Rep..

[212] Cui J.G., Zhao Y., Sethi P., Li Y.Y., Mahta A., Culicchia F., Lukiw W.J. (2009). Micro-RNA-128 (miRNA-128) down-regulation in glioblastoma targets ARP5 (ANGPTL6), Bmi-1 and E2F-3a, key regulators of brain cell proliferation. J. Neuro-Oncol..

[213] Laubscher D., Gryder B.E., Sunkel B.D., Andresson T., Wachtel M., Das S., Roschitzki B., Wolski W., Wu X.S., Chou H.-C. (2021). BAF complexes drive proliferation and block myogenic differentiation in fusion-positive rhabdomyosarcoma. Nat. Commun..

[214] Böhm M., Wachtel M., Marques J.G., Streiff N., Laubscher D., Nanni P., Mamchaoui K., Santoro R., Schafer B. (2016). Helicase CHD4 is an epigenetic coregulator of PAX3-FOXO1 in alveolar rhabdomyosarcoma. J. Clin. Investig..

[215] Shi H., Tao T., Abraham B.J., Durbin A.D., Zimmerman M.W., Kadoch C., Look A.T. (2020). ARID1A loss in neuroblastoma promotes the adrenergic-to-mesenchymal transition by regulating enhancer-mediated gene expression. Sci. Adv..

[216] Buelow D.R., Anderson J.T., Pounds S.B., Shi L., Lamba J.K., Hu S., Gibson A.A., Goodwin E.A., Sparreboom A., Baker S.D. (2020). DNA Methylation-Based Epigenetic Repression of *SLC22A4* Promotes Resistance to Cytarabine in Acute Myeloid Leukemia. Clin. Transl. Sci..

[217] Romanova E.I., Zubritskiy A.V., Lioznova A.V., Ogunleye A.J., Golotin V.A., Guts A.A., Lennartsson A., Demidov O.N., Medvedeva Y.A. (2022). RUNX1/CEBPA Mutation in Acute Myeloid Leukemia Promotes Hypermethylation and Indicates for Demethylation Therapy. Int. J. Mol. Sci..

[218] Cagnetta A., Soncini D., Orecchioni S., Talarico G., Minetto P., Guolo F., Retali V., Colombo N., Carminati E., Clavio M. (2017). Depletion of SIRT6 enzymatic activity increases acute myeloid leukemia cells’ vulnerability to DNA-damaging agents. Haematologica.

[219] Hao C., Shao X., Song J., Peng M., Lao Y., Mack R., Zhang L., Wei W., Liu N., Wang T. (2022). SIRT2 regulates proliferation and chemotherapy response of MLL-ENL-driven acute myeloid leukemia. Biochem. Biophys. Res. Commun..

[220] Poulard C., Baulu E., Lee B.H., Pufall M.A., Stallcup M.R. (2018). Increasing G9a automethylation sensitizes B acute lymphoblastic leukemia cells to glucocorticoid-induced death. Cell Death Dis..

[221] Shimizu R., Kikuchi J., Wada T., Ozawa K., Kano Y., Furukawa Y. (2010). HDAC inhibitors augment cytotoxic activity of rituximab by upregulating CD20 expression on lymphoma cells. Leukemia.

[222] Zhou X.-R., Li X., Liao L.-P., Han J., Huang J., Li J.-C., Tao H.-R., Fan S.-J., Chen Z.-F., Li Q. (2021). P300/CBP inhibition sensitizes mantle cell lymphoma to PI3Kδ inhibitor idelalisib. Acta Pharmacol. Sin..

[223] Zhang Z., Ha S.H., Moon Y.J., Hussein U.K., Song Y., Kim K.M., Park S.-H., Park H.S., Park B.-H., Ahn A.-R. (2020). Inhibition of SIRT6 potentiates the anti-tumor effect of doxorubicin through suppression of the DNA damage repair pathway in osteosarcoma. J. Exp. Clin. Cancer Res..

[224] Shinno Y., Takenobu H., Sugino R.P., Endo Y., Okada R., Haruta M., Satoh S., Mukae K., Shaliman D., Wada T. (2022). Polycomb EZH1 regulates cell cycle/5-fluorouracil sensitivity of neuroblastoma cells in concert with *MYCN*. Cancer Sci..

[225] Patties I., Jahns J., Hildebrandt G., Kortmann R.-D., Glasow A. (2009). Additive Effects of 5-Aza-2′-deoxycytidine and Irradiation on Clonogenic Survival of Human Medulloblastoma Cell Lines. Strahlenther. Onkol..

[226] Huang T., Yang Y., Song X., Wan X., Wu B., Sastry N., Horbinski C.M., Zeng C., Tiek D., Goenka A. (2021). PRMT6 methylation of RCC1 regulates mitosis, tumorigenicity, and radiation response of glioblastoma stem cells. Mol. Cell.

[227] Camero S., Vitali G., Pontecorvi P., Ceccarelli S., Anastasiadou E., Cicchetti F., Flex E., Pomella S., Cassandri M., Rota R. (2021). DNMT3A and DNMT3B Targeting as an Effective Radiosensitizing Strategy in Embryonal Rhabdomyosarcoma. Cells.

[228] Cassandri M., Pomella S., Rossetti A., Petragnano F., Milazzo L., Vulcano F., Camero S., Codenotti S., Cicchetti F., Maggio R. (2021). MS-275 (Entinostat) Promotes Radio-Sensitivity in PAX3-FOXO1 Rhabdomyosarcoma Cells. Int. J. Mol. Sci..

[229] Borcoman E., Kamal M., Marret G., Dupain C., Castel-Ajgal Z., Le Tourneau C. (2021). HDAC Inhibition to Prime Immune Checkpoint Inhibitors. Cancers.

[230] Zhong M., Gao R., Zhao R., Huang Y., Chen C., Li K., Yu X., Nie D., Chen Z., Liu X. (2022). BET bromodomain inhibition rescues PD-1-mediated T-cell exhaustion in acute myeloid leukemia. Cell Death Dis..

[231] Liu Y., Wang C., Li X., Dong L., Yang Q., Chen M., Shi F., Brock M., Liu M., Mei Q. (2021). Improved clinical outcome in a randomized phase II study of anti-PD-1 camrelizumab plus decitabine in relapsed/refractory Hodgkin lymphoma. J. Immunother. Cancer.

[232] Que Y., Zhang X.-L., Liu Z.-X., Zhao J.-J., Pan Q.-Z., Wen X.-Z., Xiao W., Xu B.-S., Hong D.-C., Guo T.-H. (2021). Frequent amplification of HDAC genes and efficacy of HDAC inhibitor chidamide and PD-1 blockade combination in soft tissue sarcoma. J. Immunother. Cancer.

[233] Bijgaart R.J.E.V.D., Kroesen M., Brok I.C., Reijnen D., Wassink M., Boon L., Hoogerbrugge P.M., Adema G.J. (2020). Anti-GD2 antibody and Vorinostat immunocombination therapy is highly effective in an aggressive orthotopic neuroblastoma model. Oncoimmunology.

[234] Grunewald C.M., Haist C., König C., Petzsch P., Bister A., Nößner E., Wiek C., Scheckenbach K., Köhrer K., Niegisch G. (2021). Epigenetic Priming of Bladder Cancer Cells With Decitabine Increases Cytotoxicity of Human EGFR and CD44v6 CAR Engineered T-Cells. Front. Immunol..

[235] Grimm J., Bhayadia R., Gack L., Heckl D., Klusmann J.-H. (2022). Combining LSD1 and JAK-STAT inhibition targets Down syndrome-associated myeloid leukemia at its core. Leukemia.

[236] Alcitepe I., Salcin H., Karatekin I., Kaymaz B.T. (2022). HDAC inhibitor Vorinostat and BET inhibitor Plx51107 epigenetic agents’ combined treatments exert a therapeutic approach upon acute myeloid leukemia cell model. Med. Oncol..

[237] Ciceri S., Carenzo A., Iannó M.F., Bertolotti A., Morosi C., Luksch R., Spreafico F., Collini P., Radice P., Massimino M. (2022). Gene expression-based dissection of inter-histotypes, intra-histotype and intra-tumor heterogeneity in pediatric tumors. Sci. Rep..

[238] Groves A., Clymer J., Filbin M.G. (2022). Bromodomain and Extra-Terminal Protein Inhibitors: Biologic Insights and Therapeutic Potential in Pediatric Brain Tumors. Pharmaceuticals.

[239] Bolin S., Borgenvik A., Persson C.U., Sundström A., Qi J., Bradner J.E., Weiss W.A., Cho Y.-J., Weishaupt H., Swartling F.J. (2018). Combined BET bromodomain and CDK2 inhibition in MYC-driven medulloblastoma. Oncogene.

[240] Bharathy N., Berlow N.E., Wang E., Abraham J., Settelmeyer T.P., Hooper J.E., Svalina M.N., Bajwa Z., Goros M.W., Hernandez B.S. (2019). Preclinical rationale for entinostat in embryonal rhabdomyosarcoma. Skelet. Muscle.

[241] Pan T., Qi J., You T., Yang L., Wu D., Han Y., Zhu L. (2018). Addition of histone deacetylase inhibitors does not improve prognosis in patients with myelodysplastic syndrome and acute myeloid leukemia compared with hypomethylating agents alone: A systematic review and meta-analysis of seven prospective cohort studies. Leuk. Res..

[242] Daskalakis M., Brocks D., Sheng Y.-H., Islam S., Ressnerova A., Assenov Y., Milde T., Oehme I., Witt O., Goyal A. (2018). Reactivation of endogenous retroviral elements via treatment with DNMT- and HDAC-inhibitors. Cell Cycle.

[243] Okoye-Okafor U.C., Javarappa K.K., Tsallos D., Saad J., Yang D., Zhang C., Benard L., Thiruthuvanathan V.J., Cole S., Ruiz S. (2022). Megakaryopoiesis impairment through acute innate immune signaling activation by azacitidine. J. Exp. Med..

[244] Iltar U., Alhan F.N., Vural E., Ataş Ü, Sözel H., Doğan Ö, Boduroğlu A., Yücel O.K., Salim O., Ündar L. (2021). Recurrent arthritis as an unexpected side effect associated with azacitidine in a patient with myelodysplastic syndrome. J. Oncol. Pharm. Pr..

[245] da Mata M.F., Martins M.V., Rato J., Madeira M., Gonçalves J.-P., Teixeira A., Anjos R. (2022). Azacitidine-induced massive pericardial effusion in a child with myelodysplastic syndrome. J. Oncol. Pharm. Pr..

[246] Goldberg J., Sulis M.L., Bender J., Jeha S., Gardner R., Pollard J., Aquino V., Laetsch T., Winick N., Fu C. (2020). A phase I study of panobinostat in children with relapsed and refractory hematologic malignancies. Pediatr. Hematol. Oncol..

[247] Campbell C.T., Haladyna J.N., Drubin D.A., Thomson T.M., Maria M.J., Yamauchi T., Waters N.J., Olhava E.J., Pollock R.M., Smith J.J. (2017). Mechanisms of Pinometostat (EPZ-5676) Treatment–Emergent Resistance in *MLL*-Rearranged Leukemia. Mol. Cancer Ther..

[248] Marcos-Villar L., Nieto A. (2019). The DOT1L inhibitor Pinometostat decreases the host-response against infections: Considerations about its use in human therapy. Sci. Rep..

